# Autophagy Induction via STING Trafficking Is a Primordial Function of the cGAS Pathway

**DOI:** 10.1038/s41586-019-1006-9

**Published:** 2019-03-06

**Authors:** Xiang Gui, Hui Yang, Tuo Li, Xiaojun Tan, Peiqing Shi, Minghao Li, Fenghe Du, Zhijian J. Chen

**Affiliations:** 1 Department of Molecular Biology, University of Texas Southwestern Medical Center, Dallas, TX 75390-9148; 2 Howard Hughes Medical Institute, University of Texas Southwestern Medical Center, Dallas, TX 75390-9148

## Abstract

Cyclic GMP-AMP (cGAMP) synthase (cGAS) detects infections or tissue damage by binding to microbial or self-DNA in the cytoplasm^1^. Upon binding DNA, cGAS produces cGAMP that binds to and activates the adaptor protein STING, which then activates the kinases IKK and TBK1 to induce interferons and other cytokines^2–6^. Here, we report that STING also activates autophagy through a mechanism independent of TBK1 activation and interferon induction. Upon binding cGAMP, STING translocates to the ER-Golgi intermediate compartment (ERGIC) and the Golgi in a process dependent on the COP-II complex and ARF GTPases. STING-containing ERGIC serves as a membrane source for LC3 lipidation, a key step in autophagosome biogenesis. cGAMP induced LC3 lipidation through a pathway dependent on WIPI2 and ATG5 but independent of the ULK and VPS34/BECLIN kinase complexes. Furthermore, we show that cGAMP-induced autophagy is important for the clearance of DNA and viruses in the cytosol. Interestingly, STING from the sea anemone *Nematostella vectensis* induces autophagy but not interferons in response to stimulation by cGAMP, suggesting that induction of autophagy is a primordial function of the cGAS-STING pathway.

To study DNA-induced autophagy, we transfected interferon-stimulatory DNA (ISD) into BJ cells, which are human fibroblasts immortalized by the telomerase hTERT. DNA transfection not only activated downstream components of the cGAS pathway such as STING, TBK1, and IRF3 but also stimulated conversion of LC3 into a lipidated form (LC3-II) (Extended Data Fig. 1a). In contrast, the synthetic double-stranded RNA analogue poly(I:C) failed to stimulate LC3 lipidation despite stimulating phosphorylation of TBK1 and IRF3. Similarly, infection with the DNA virus herpes simplex virus-1 (HSV-1), but not the RNA virus Sendai virus, stimulated LC3 lipidation (Extended Data Fig. 1b). Furthermore, cGAMP delivery into BJ cells was sufficient to induce robust LC3 lipidation and STING degradation (Extended Data Fig. 1a), which was more prominent when the cells were pretreated with chloroquine, suggesting an increase of the autophagy flux after cGAMP stimulation (Extended Data Fig. 1c). cGAMP-stimulated autophagosome formation was also visualized by GFP-LC3 live cell imaging (Supplementary Video 1). Electron microscopy demonstrated that cGAMP induced the formation of double-membrane autophagosomes (Fig. 1a, b). Loss of cGAS blocked LC3 conversion triggered by herring testis (HT) DNA but not cGAMP, whereas loss of STING blocked LC3 conversion induced by both HT-DNA and cGAMP (Fig. 1c). Interestingly, BJ cells deficient in TBK1 still retained LC3 lipidation in response to cGAMP treatment (Fig. 1d). Additional experiments showed that STING vesicle trafficking but not TBK1 phosphorylation of STING at Ser366 is required for cGAMP-induced autophagy (Extended Data Fig. 1d–1l; Supplementary Videos 2 and 3; Supplementary Information).

Next, we delineated the domains within STING required for autophagy induction. Deletion of the C-terminal activation domain (STING 1–340) abolished phosphorylation of TBK1 and IRF3 but did not impair LC3 conversion or STING degradation induced by cGAS or cGAMP, furthermore, a mutation of a cGAMP-binding residue (R238A) in STING 1–340 blocked STING degradation and LC3 conversion (Fig. 1e). Further mapping identified a small region spanning residues 330–334 of STING as important for autophagy induction (Extended Data Fig. 2a, b). Point mutations of residues within this region revealed that mutation of L333 and R334 to alanine within full-length STING abrogated LC3 conversion, phosphorylation of TBK1 and IRF3, and formation of STING and LC3 puncta induced by cGAMP (Extended Data Fig. 2c, d). The sequence surrounding this ‘LR’ motif is evolutionarily conserved (Extended Data Fig. 2e) and is found in STING of *Nematostella vectensis* (nvSTING), an anemone species that possesses a functional cGAS-STING pathway^7^. nvSTING does not contain the C-terminal domain required for activation of the type-I interferon pathway found in vertebrate STING (Extended Data Fig. 2e). nvcGAS was found to produce 3’3’-cGAMP^7^; however, we found that 2’3’-cGAMP stimulated stronger LC3 conversion than 3’3’-cGAMP in HEK293T cells stably expressing human STING or nvSTING, but neither compounds stimulated TBK1 activation through nvSTING (Fig. 1f). Truncated human STING (1–340) mimicked nvSTING function after 2’3’-cGAMP stimulation in triggering LC3 lipidation without TBK1 or IRF3 phosphorylation (Extended Data Fig. 3a). Additional experiments showed that nvcGAS produces 2’3’-cGAMP rather than 3’3’-cGAMP (Extended Data Fig. 3b, 3c and 3d; Supplementary Information). STING from *Xenopus tropicalis* also lacks the C-terminus required for TBK1 and IRF3 activation (Extended Data Fig. 3e). In response to 2’3’-cGAMP stimulation, Xenopus STING translocated from the ER and triggered LC3 conversion without activating TBK1 or IRF3 (Extended Data Fig. 3f–h). In contrast, STING from *Danio rerio*, which contains a C-terminal activation domain homologous to that of human STING, stimulated LC3 conversion as well as phosphorylation of TBK1 and IRF3 (Extended Data Fig. 3f–h). Taken together, our data show that the autophagy-inducing but not the interferon-inducing function of vertebrate STING is conserved in Nematostella and Xenopus, suggesting autophagy induction is a primordial function of the cGAS-STING pathway.

To investigate the mechanism by which STING activates autophagy, we examined STING trafficking using confocal microscopy. Upon cGAMP stimulation, STING first colocalized at the perinuclear region with ERGIC-53, a marker of the ER Golgi intermediate compartment (ERGIC), before colocalizing with GFP-LC3-positive autophagosomes (Extended Data Fig. 2a and Supplementary Video 4). STING trafficking to the ERGIC was also blocked by Golgicide A (GCA; Extended Data Fig. 4b). These results are intriguing because recent studies have suggested that the ERGIC is a major source of membranes for starvation-induced LC3 lipidation^8^. To test if the ERGIC serves as a membrane source for cGAMP-induced LC3 lipidation, we performed an in vitro reconstitution assay using cytosol (S100) from WT HEK293T cells and membranes (P25) from *ATG5*^−/−^ HEK293T-STING cells stimulated by cGAS transfection (Extended Data Fig. 4c). Consistent with a previous report^8^, LC3 conversion was detected 1 hour after membranes from *ATG5*^−/−^ cells were incubated with cytosol from starved WT cells (Extended Data Fig. 4d). Importantly, LC3 conversion was detected when membranes from cGAS-stimulated *ATG5*^−/−^ cells were incubated with cytosol from unstimulated WT cells (Fig. 2a and Extended Data Fig. 4d). In contrast, membranes from cGAS-stimulated cells treated with BFA lost the ability to stimulate LC3 conversion (Fig. 2b). These results suggest that vesicles budding from the ER and/or Golgi in cGAS-stimulated cells serve as the membrane source for LC3 conjugation by the cytosolic ATG5 conjugation machinery. To determine the types of vesicles that stimulate LC3 conjugation, we further fractionated the membranes from cGAS-stimulated *ATG5*^−/−^ HEK293T-STING cells by differential centrifugation followed by sucrose gradient ultracentrifugation and OptiPrep™ (iodixanol) density gradient ultracentrifugation (Extended Data Fig. 4c). Each membrane fraction was incubated with the cytosolic extract from HEK293T cells to test for the ability to support LC3 conjugation (Fig. 2c and Extended Data Fig. 4e, f). This analysis revealed that membrane fractions enriched in STING and the ERGIC markers ERGIC-53 and Sec22b had enhanced activity in stimulating LC3 conversion, suggesting that the ERGIC serves as a membrane source for LC3 conjugation. Additional experiments showed that STING traffics to lysosomes via autophagosomes and endosomes in a Rab7a-dependent manner (Extended Data Figure 5; Supplementary Information).

We tested the role of several autophagy-related genes in cGAMP-induced LC3 lipidation and STING degradation. ATG5 deficiency abolished LC3 lipidation but not STING degradation (Extended Data Fig. 6a). Basal levels of LC3 lipidation appeared to be higher in ATG9 deficient cells, but cGAMP-induced STING degradation was normal in these cells (Extended Data Fig. 6b). We next examined whether STING induces the inhibition of mTOR, which is known to regulate autophagy. mTOR and 4E-BP1 were dephosphorylated after treatment with the mTOR inhibitor Torin 1 or rapamycin, but not after HT-DNA or cGAMP treatment, which triggered more robust LC3 conversion than the mTOR inhibitors (Extended Data Fig. 6c). WIPI2 is a phosphotidylinositol-3-phosphate (PI3P) effector protein important for LC3 lipidation in the conventional autophagy pathway^9^. We found that LC3 lipidation and P62 degradation were diminished in WIPI2-deficient cells after HT-DNA or cGAMP treatment while TBK1 and IRF3 phosphorylation as well as STING degradation remained unaffected (Fig. 2d), suggesting that STING may induce LC3 conjugation through WIPI2. The kinases ULK1 and ULK2 are required for conventional autophagy induced by Torin 1 (Fig. 2e and Extended Data Fig. 6d). Interestingly, *Ulk1*^*−/−*^*Ulk2*^*−/−*^ MEFs showed normal STING degradation, LC3 conversion, and double-membrane autophagosome formation after HT-DNA transfection, cGAMP delivery, or DMXAA treatment (Fig. 2e and Extended Data Fig. 6e, f). Similarly, cGAMP-induced LC3 lipidation and autophagosome formation were normal in the absence of Beclin 1, which is required for conventional autophagy (Fig. 2f and Extended Data Fig. 7; see Supplementary Information). These results suggest that STING-containing ERGIC vesicles may activate LC3 lipidation through a WIPI2- and ATG5-dependent mechanism that is distinct from conventional autophagy.

Trafficking of proteins from the membrane and lumen of the ER is initiated by the budding of vesicles that requires the GTPase SAR1A and the COP-II complex that includes SEC24^15,16^. We found that knockdown of SAR1A or SEC24C expression in HeLa cells by siRNA blocked STING puncta formation induced by cGAMP (Extended Data Fig. 8a, b). Depletion of each protein also inhibited induction of IFNβ and the chemokine CXCL10 by DNA or cGAMP but not by poly(I:C) (Fig 3a, b and Extended Data Fig. 8c, d). Co-immunoprecipitation experiments showed that cGAMP induced the interaction between STING and SEC24C at early time points and that this interaction was disrupted by mutating L333 and R334 on STING (Fig. 3c). We then used the CRISPR technology to knock out SEC24C in HEK293T-STING cells; a single-guide RNA (sgRNA) against SEC24C was efficient in nearly depleting endogenous SEC24C without single cell cloning (Fig. 3d). Depletion of SEC24C inhibited LC3 conversion and phosphorylation of TBK1 and IRF3; the residual activity may be due to the presence of some WT cells in the pool. When the knockout cells were rescued with Sec24C, the cGAMP signaling pathway was fully restored (Fig. 3d). These results indicate that the formation of the COP-II vesicle is important for the signaling events downstream of cGAMP binding to STING. We further showed that cGAMP stimulation enhanced the binding of the GTPase ARF1, which regulates vesicle trafficking^15,17^, to its effector protein GGA3 (Fig. 3e), indicating that cGAMP activates ARF1. Inhibition or depletion of ARF1 strongly suppressed the signaling cascade induced by DNA but not RNA (Extended Data Fig. 8e–i; Supplementary Information).

Our findings that the autophagy-inducing activity of STING predates its interferon-inducing activity during evolution raises the question of the role of autophagy induction by the cGAS-STING pathway. We first examined whether autophagy induction by cytosolic DNA provides a mechanism for DNA clearance from the cytosol. Cy3-ISD activated endogenous cGAS to produce cGAMP, which led to LC3 puncta formation in STING-expressing but not STING-deficient cells (Extended Data Fig. 9a). Co-localization of LC3 puncta with Cy3-ISD was evident in cells expressing WT STING but not in those expressing mutant STING (R238A/Y240A) (Fig. 4a, and Extended Data Fig. 9a), suggesting DNA was targeted by STING-induced autophagy. Live cell imaging showed that Cy3-ISD was enclosed by the LC3 puncta (Supplementary Video 5) and cGAMP delivery further accelerated LC3 puncta formation and DNA degradation (Supplementary Video 6). These results indicate that cGAMP-induced autophagy facilitates the clearance of cytosolic DNA. To investigate whether cGAMP enhances the clearance of endogenous DNA, we used arabinofuranosyl cytidine (Ara-C), which causes DNA damage by interfering with DNA synthesis. Ara-C treatments led to detection of DNA in the cytosol by an antibody against dsDNA (Fig. 4b). cGAMP treatment resulted in the disappearance of cytosolic DNA, an effect blocked by GCA (Fig. 4b). We further tested whether cGAMP-induced autophagy is important for host defense against viruses. Quantitative PCR (qPCR) measurement of the viral genome equivalents (VGE) revealed that cells expressing full-length STING or STING (1–340), but not STING (1–340) with a cGAMP binding mutation (R238A/Y240A), had reduced HSV-1 titer in response to cGAMP treatment (Fig. 4c). Similarly, Fluorescence activated cell sorting (FACS) analyses showed that the titers of HIV-GFP (Extended Data Fig. 9b) and HSV-GFP virus (Extended Data 9c) were significantly lower in cGAMP-stimulated HEK293T cells expressing full-length STING or STING(1–340), but not the STING (1–340, R238A/Y240A) mutant. STING (1–340) did not induce IFNβ or TNFα in response to cGAMP delivery, confirming that the antiviral effect of this STING mutant is independent of cytokine induction (Extended Data Fig. 9d). To further evaluate the role of autophagy in antiviral defense, we employed the *Legionella* protein RavZ, an enzyme that irreversibly cleaves LC3 from phosphatidylethanolamine on the membrane^18^. WT RavZ expression in HEK293T-STING cells removed LC3 conjugation (Extended Data Fig. 9e) and largely obliterated the inhibitory effect of cGAMP on HSV-1 replication (Fig. 4d); the residual inhibitory effect of cGAMP may be due to induction of interferons and other antiviral cytokines. In contrast, a catalytically inactive mutant of RavZ (C258A) did not interfere with the inhibition of HSV-1 replication by cGAMP (Fig. 4d and Extended Data Fig. 9e). Knocking out ATG5 but not TBK1 or BCLN1 largely abrogated the inhibitory effect of cGAMP on HSV-1 as measured by HSV-GFP fluorescence intensity and normalized virus titer (Extended Data Fig. 9f, g). Taken together, these results indicate that cGAMP-induced autophagy plays a crucial role in antiviral defense.

A unique and important feature of the cGAS-STING pathway is the robust activation of autophagy in addition to induction of interferons and inflammatory cytokines. cGAMP-induced LC3 lipidation is independent of TBK1 and the C-terminal signaling domain of STING, which is required for type-I interferon induction^19,20^. Conversely, activation of TBK1 and IRF3 remains intact in ATG5-deficient cells that are defective in LC3 lipidation and autophagosome formation. Thus, the autophagy- and interferon-inducing activities of STING can be uncoupled. After cGAMP binding to STING, STING interacts with SEC24C and buds from the ER into COP-II vesicles, which then form the ERGIC (Extended Data Fig. 10). The ERGIC serves as the membrane source for WIPI2 recruitment and LC3 lipidation, leading to formation of autophagosomes that target cytosolic DNA or DNA viruses for degradation by the lysosome. A fraction of STING traffics from the ERGIC to the Golgi network and post-Golgi vesicles such as late endosomes, where STING activates TBK1 and IRF3, leading to type-I interferon induction. STING in the autophagosomes and endosomes continues to traffic to the lysosome where it is degraded in a RAB7-dependent manner (Extended Data Fig. 10). Remarkably, the sea anemone, which predates *Homo sapiens* by more than 500 million years, possesses a STING homologue (nvSTING) lacking the C-terminal TBK1 activation domain^7^; nevertheless, nvSTING is still capable of stimulating LC3 conversion in response to cGAMP. Thus, autophagy induction is an ancient and highly conserved function of the cGAS-STING pathway that predates the emergence of the type-I interferon pathway in vertebrates.

## METHODS

### Reagents and General Methods

2’3’-cGAMP was synthesized as previously described^21^. Poly (I:C), herring testis (HT) DNA, Ara-C, and Aphidicolin were from Sigma-Aldrich. ISD and CY3-ISD were prepared from equimolar amounts of sense and antisense DNA oligonucleotide (sense: 5-TACAGATCTACTAGTGATCTATG-3; anti-sense: 5-ACTGATCTGTACATGATCTACA-3). The oligonucleotides, synthesized at Sigma-Aldrich, were heated at 95°C for 5 min and cooled to room temperature. Brefeldin A, golgicide A, BX-795, TPCA-1, MG132, and Velcade were purchased from Selleckchem; Bafilomycin A1 and chloroquine were from Invivogen. siRNA oligos were purchased from Sigma and transfected into cells using Lipofectamine RNAiMAX (Thermo Fisher Scientific). The sense strand sequences are shown in Table S1.

The procedures for IRF3 dimerization assay, SDS-PAGE, Western blotting, and immunoprecipitation have been described previously^22^. cGAMP was delivered into cells by permeabilization with digitonin (10 μg/ml) for 15 min in buffer A (50 mM HEPES-KOH, pH 7.2, 100 mM KCl, 3 mM MgCl_2_, 0.1mM DTT, 85 mM Sucrose, 0.2% BSA, 1mM ATP). The concentration of cGAMP used in stimulating BJ cells was 0.2 μM or 0.5 μM unless indicated otherwise. ISD, HT-DNA, and Poly (I:C) were transfected into cells using lipofectamine 2000 (Thermo Fisher) at a concentration of 2 μg/ml. CY3-ISD (1 μg/ml) was delivered into cells by permeabilization with perfringolysin O (PFO; 0.1 μg/ml). 1 hour pretreatment was used for all inhibitors before DNA transfection or cGAMP stimulation at the following concentrations: Brefeldin A, 2 μM; Bafilomycin A1, 0.2 μM; Chloroquine, 20 μM; MG132, 10 μM; Velcade, 2 μM; Golgicide A, 10 μM.

### Antibodies

The rabbit polyclonal antibodies against human STING were described previously^23^. Rabbit antibodies against mouse STING, p-IRF3(Ser396), p-TBK1(Ser172), p-IKKβ(Ser177), ATG5, ATG9, Beclin1, calreticulin, and GAPDH were from Cell Signaling. Mouse antibody against STING was purchased from R&D Systems; rabbit antibodies against human IRF3, TGN38, and ARF1 and mouse antibody against CD63 were from Santa Cruz Biotechnology; rabbit antibody against LC3 was from Novus Biologicals; mouse antibodies against P62 and GGA3 were from BD Transduction Laboratories; mouse antibody against ERGIC-53 was from Axxora; rabbit antibody against Sec22b was from Synaptic Systems; mouse antibody against Flag tag, rabbit antibodies against ERGIC53, and β-tubulin, anti-Flag (M2)-conjugated agarose and anti-HA–conjugated agarose were from Sigma; HA antibody were from Covance; rabbit antibodies against GBF1, LAMP2, and Giantin were from Abcam; rabbit antibody against beta-COP was from Thermo Fisher; rabbit antibodies against ARFGEF1, ARFGEF2, and SEC24C were from Bethyl Laboratories.

### Expression constructs, Viruses, Cells, and Transfection

For transient expression in mammalian cells, human cDNAs encoding N-terminal tagged cGAS and C-terminal tagged STING were cloned into pcDNA3. For stable expression in mammalian cells, human cDNAs encoding C-terminal Flag-tagged STING and its mutants were cloned into a pTY-EF1A-IRES lentiviral vector^24^, which was modified from PTY-shRNA-EF1a-puroR-2a-Flag provided by Dr Yi Zhang (Harvard Medical School). These lentiviruses were packaged in HEK293T cells and transduced into target cells as described previously^23^. STING mutants were constructed using the QuikChange Site-Directed Mutagenesis Kit. Plasmids for mammalian expression of WT and C258A RavZ were kindly provided by Dr. Craig Roy (Yale). Plasmids and HT-DNA were transfected into cells using Lipofectamine 2000 (Life Technologies).

All cells were cultured at 37°C in an atmosphere of 5% (v/v) CO_2_. HEK293T and HeLa cells were cultured in Dulbecco’s modified Eagle’s medium (DMEM) supplemented with 10% (v/v) cosmic calf serum (Hyclone), penicillin (100 U/ml), and streptomycin (100 μg/ml). MEF, L929, and BJ-hTERT cells were cultured in DMEM supplemented with 10% (v/v) fetal bovine serum (FBS, Atlanta) and antibiotics. THP1 cells were cultured in RPMI 1640 supplemented with 10% FBS, 2 mM β-mercaptoethanol, and antibiotics. Hela cells stably expressing GFP-LC3 and mouse bone marrows containing macrophages deficient in Beclin1 were provided by Dr. Beth Levine (UT Southwestern). Ulk1^−/−^Ulk2^−/−^ (DKO) MEF cells were from Sigma (14050803–1VL). To induce autophagy by starvation, cells were washed with PBS three times and cultured in Earles Balanced Salt Solution (EBSS).

Sendai virus (Cantell strain, Charles River Laboratories) was used at a final concentration of 50 hemagglutinating units/ml. HSV-1 WT strain was propagated and titered by plague assays on Vero Cells and used at the indicated multiplicity of infection (MOI) in BJ cells. The HSV1 ΔICP34.5 strain was used at the indicated MOI in BJ and HEK293T cells. Plasmids for HIV-GFP and VSV-G have been described previously^25^; HIV-GFP lentiviral plasmid was co-transfected with the VSV-G plasmid into HEK293T cells for virus packaging. Supernatants containing the viruses were harvested, filtered, and concentrated by PEG8000 precipitation. The titers of HIV-GFP virus were measured by infecting HEK293T cells and performing flow cytometry analysis of GFP^+^ cells 24 hr after infection in the presence of 10 μg/mL polybrene. HSV1-GFP was provided by Dr. Akiko Iwasaki (Yale).

### Generation of Knockout Cells by CRISPR/Cas9

Single-guide RNAs (sgRNA) were designed to target the human cGAS, STING, TBK1, ARF1, GBF1, ATG5, ATG9, ULK1, BECN1, and SEC24C genomic loci (Table S2). The sgRNA sequence driven by a U6 promoter was cloned into a lentiCRISPR vector that also expresses Cas9 as previously described^26^. The lentiviral plasmid DNA was then packaged into a lentivirus for infection in HEK293T cells or BJ cells. Infected cells were selected in puromycin (2 μg/ml) for 2 weeks before single colonies were chosen and tested by immunoblotting, TA cloning, and DNA sequencing.

### Generation of Primary Mouse Embryonic Fibroblasts (MEFs) and Bone Marrow Derived Macrophages (BMDM)

cGas^−/−^ mice were generated as described previously^27^. *Sting*^*gt/gt*^ mice were from the Jackson laboratory^28^. These strains were maintained on a C57BL/6J background. MEFs were generated from E13.5 embryos of WT and mutant mice under normal culture conditions^29^. Fresh leg bones from LysM-Cre^+^Beclin 1^flox/flox^ and Beclin 1^flox/flox^ mice were provided by Dr Beth Levine (UT Southwestern). BMDMs were generated as described previously^27^. All mice were bred and maintained under specific pathogen-free conditions in the animal care facility of University of Texas Southwestern Medical Center at Dallas according to experimental protocols approved by the Institutional Animal Care and Use Committee.

### Immunostaining, Confocal Microscopy, and Live Cell Imaging

For immunostaining, cells were fixed with 4% paraformaldehyde, permeabilized with Triton X100 (0.2%), and stained with a primary antibody followed by a fluorescent secondary antibody. Nuclei were labeled by staining with DAPI in the mounting medium (Vectashield). Images of cells were collected with a Zeiss LSM710 META laser scanning confocal microscope and processed using Zeiss LSM image browser. In some experiments, images were collected with a Nikon A1R confocal microscope and processed using ImageJ. For live cell imaging, cells were grown on a four-chambered cover glass (Lab-Tek II, 155382) at a density of 40,000 cells per chamber (~50% confluency) in 5% CO_2_ and 20% O_2_ at 37 °C, and videos were recorded using Nikon A1R confocal laser microscope system and further processed and analyzed using ImageJ.

### Flow Cytometry

After HIV-GFP or HSV-GFP infection, cells were washed in FACS buffer (PBS, 1% BSA), fixed with 2% paraformaldehyde, and analyzed on BD FACS Calibur (BD Biosciences). Data analysis was performed using FlowJo software.

### Electron Microscopy

BJ cells were grown on glass bottom plates before stimulation with cGAMP (0.2μM for BJ cells and 1μM for MEF cells and HEK293T cells) or Torin1 (1μM for all cells). Samples were fixed, sectioned, stained, and coated by the UTSW Electron Microscopy core facility. The images were visualized using FEI Tecnai™ transmission electron microscopes (TEMs).

### RT-qPCR and HSV1 genome qPCR

Reverse transcription quantitative PCR (RT-qPCR) reactions were carried out using the iScript cDNA synthesis kit and iQ SYBR Green Supermix (Bio-Rad). qPCR was performed on an Applied Biosystems Vii7 using the primers shown in Table S1. HSV-1 infected cells were washed and lysed in a buffer containing 1% SDS, 50mM Tris-CL (pH 7.5), and 10mM EDTA, and the cell extract was incubated with proteinase K (2 mg/mL) at 37 °C for 30 min. DNA was extracted via phenol/chloroform extraction and ethanol precipitation. Viral DNA was quantified by qPCR using three different pair of primers corresponding to distinct regions of the HSV-1 genome (Table S3).

### In vitro LC3 lipidation assay

The in vitro LC3 assay was modified from published methods^30^. Cytoplasmic extract (S100) was prepared from WT or *ATG5*^*−/−*^ HEK293T cells grown in normal or EBSS starvation media for 2 hr. After washing with PBS, cells were lysed by passing through a 25 G needle in a 3x cell pellet volume of hypotonic buffer (20 mM HEPES-KOH, pH 7.2, 10 mM KCl, 3 mM MgCl_2_) plus cocktail protease inhibitors and phosphatase inhibitors (Roche). The cell lysate was centrifuged at 100,000×g for 2 hr to collect the S100 supernatant. For P25 membrane preparation, *ATG5*^*−/−*^ HEK293T cells were either left untreated or transfected with a cGAS expression plasmid for 12 hr. Then, cells were washed with PBS and homogenized by douncing for 20 times in a buffer (20 mM HEPES-KOH, 400 mM sucrose, 0.5 mM EDTA). The homogenate was centrifuged at 1000 × g for 5 min to remove cell debris and nuclei. The supernatant (S1) was further centrifuged at 5,000 × g for 10 min to precipitate mitochondria and other heavy organelles (P5). The supernatant (S5) was further centrifuged at 25,000 × g for 30 min to pellet membranes (P25). For each reaction, S100 (2 mg/ml final concentration), ATP regeneration system (40 mM creatine phosphate, 0.2 mg/ml creatine phosphokinase, and 1 mM ATP), GTP (0.15 mM), P25 or different membrane fractions (0.2 mg/ml) were incubated in a final volume of 30 μl. The mixture was incubated at 30°C for the indicated time followed by SDS-PAGE and immunoblotting.

### Membrane Fractionation

#### (A) Differential centrifugation of membranes.

Cells (five 15-cm dishes) were cultured to confluence, harvested, and homogenized by passing through a 25 G needle ten times in a 5x cell pellet volume of hypotonic buffer. Homogenates were subjected to sequential centrifugation at 1,000×g (10 min), 5,000×g (10 min), 25,000×g (20 min), and 100,000×g (30 min) to collect the P1, P5, P25, and P100 membranes, respectively. Membrane fractions containing equal amounts of proteins were used for the LC3 lipidation assay as described above.

#### (B) Sucrose gradient ultracentrifugation.

The P25 membrane fraction, which contained the highest LC3 lipidation activity, was used to purify ERGIC- and Golgi-containing fractions using a Golgi isolation kit (Sigma). The P25 membranes were suspended in 0.75 ml of 1.25 M sucrose buffer and overlaid first with 0.5 ml of 1.1 M and then with 0.5 ml of 0.25 M sucrose buffer and centrifuged at 120,000×g for 3 hr. The P25 L fraction at the interface between the 0.25 M and 1.1 M sucrose layers and the pellet on the bottom (P25 P fraction) were used to test LC3 lipidation activity.

#### (C) OptiPrep gradient ultracentrifugation.

The P25 L fraction was suspended in 1 ml 19% OptiPrep for the following OptiPrep step gradient from bottom to top: 0.33 ml 22.5%, 0.66 ml 19% (sample), 0.6 ml 16%, 0.6 ml 12%, 0.66 ml 8%, 0.33 ml 5%, and 0.14 ml 0%. Each density of OptiPrep was prepared by diluting 50% OptiPrep (20 mM Tricine-KOH, pH 7.4, 42 mM sucrose and 1 mM EDTA) with a buffer of 20 mM Tricine-KOH, pH 7.4, 250 mM sucrose, and 1 mM EDTA. The OptiPrep gradient was formed by centrifugation using a SW60 Swinging bucket Ti Rotor at 150,000×g for 3 hr with ten fractions collected from the top to bottom. Fractions were diluted with hypotonic buffer and membranes were collected by centrifugation at 100,000×g for 1 hr. The activity of each fraction was tested as described before^30^.

### cGAMP detection by Mass Spectrometry

293T cells were transfected with expression plasmids encoding cGAS, DncV, or nvcGAS. Small molecules were extracted from cells as described previously^31^. Briefly, cells were lysed in 80% methanol and 2% acetic acid solution before the addition of an internal standard and an equal volume of 2% acetic acid. Insoluble fractions were pelleted by centrifugation and were extracted two more times in 2% acetic acid. After combining all three extracts, cyclic dinucleotides were enriched by solid phase extraction on a Hypersep NH2 column (Thermo), washed with 2% acetic acid and with 80% methanol, and eluted with 20% ammonium hydroxide in methanol. After drying by vacuum centrifugation, samples were reconstituted in water and analyzed by a Dionex U3000 HPLC coupled with a TSQ Quantiva Triple Quandrupole mass spectrometer. Data was collected by product ion scans that target a m/z of 675 and analyzed with XCalibur (Thermo).

### Statistics and reproducibility

Representative results from at least two independent repeats are shown for every figure except where specified otherwise in the figure legends.

Data are shown as the mean ± SEM. unless indicated otherwise. GraphPad Prism 8 software (GraphPad) was applied for statistical analysis. For comparisons between 2 groups, the Student’s *t*-test (unpaired and 2-tailed) was applied.

### Data Availability

The authors declare that all relevant data supporting the findings of this study are available within the paper and its supplementary information files. Additional information including raw data is available from the corresponding author upon reasonable request.

## Extended Data

**Extended Data Figure 1.**
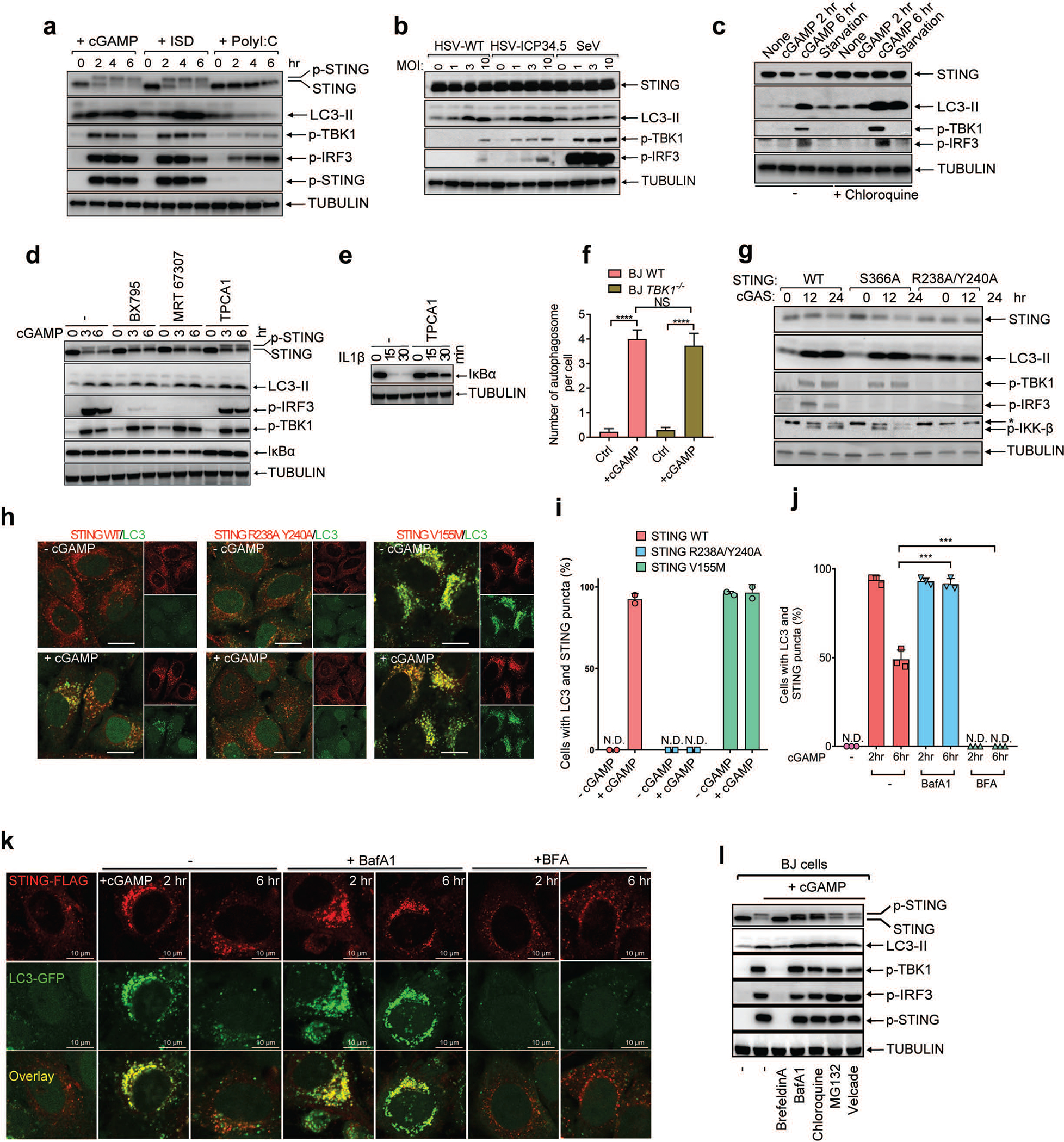
cGAMP-induced LC3 lipidation requires vesicle trafficking but not TBK1 or IKK. **a,** DNA and cGAMP but not RNA trigger LC3 lipidation. BJ cells were stimulated with cGAMP by digitonin permeabilization or transfected with ISD or poly (I:C). Cell lysates were analyzed by immunoblotting with the indicated antibodies. **b,** DNA virus but not RNA virus induces LC3 conversion. BJ cells were infected with wild type (WT) HSV-1, HSV-1 ΔICP34.5, or Sendai virus (SeV) at the indicated multiplicity of infection (MOI) for 6 hr followed by immunoblotting. **c,** cGAMP induces STING degradation in the lysosome. HeLa cells stably expressing STING-Flag were treated with cGAMP or starved in the presence or absence of chloroquine, followed by immunoblotting. **d,** Inhibition of TBK1 or IKK does not impair LC3 lipidation. Inhibitors of TBK1 (BX-795 or MRT 67307) or IKK (TPCA1) were incubated with BJ cells before stimulation of the cells with cGAMP. Cell lysates were analyzed by immunoblotting. **e,** Control experiment showing that TPCA1 inhibits IκBα degradation (by inhibiting IKK). **f,** Quantification of double-membrane autophagosomes in BJ WT and BJ *TBK1*^*−/−*^ cells. The cells were stimulated with cGAMP as indicated. Quantification was shown as the number of double-membrane autophagosomes per cell by counting on BJ cells (n=13, 12, 17, 11). Mean ±SEM are shown. **** p < 0.0001 (two-tailed Student’s *t*-test); NS, not significant (significance level, α = 0.01). **g,** STING S366 phosphorylation by TBK1 is essential for IRF3 phosphorylation but not LC3 conversion. HEK293T cells stably expressing WT or mutant STING (S366A or R238/Y240A) were transfected with a cGAS expression plasmid followed by immunoblotting. **h,** Hela cells stably expressing GFP-LC3 and different STING mutants (R238A Y240A or V155M) were stimulated with cGAMP followed by confocal immunofluorescence microscopy. **i,** Quantification of the cells with colocalization of LC3 and STING puncta. The percentage of cells with colocalized LC3 and STING was quantified from 100 cells (n = 2). N.D., not detectable. **j&k,** HeLa cells stably expressing GFP-LC3 and STING-Flag were treated with BafA1 or BFA followed by stimulation with cGAMP and confocal immunofluorescence microscopy. Quantification of the cells with colocalization of LC3 and STING puncta. The percentage of cells with colocalized LC3 and STING was quantified from 100 cells (n = 3, mean ±SD, two-tailed Student’s *t*-test). N.D., not detectable. **l,** BJ cells were treated with brefeldin A (BFA), lysosome inhibitors (bafilomycin A1 [BafA1] or chloroquine), or proteasome inhibitors (MG132 or Velcade) before stimulation with cGAMP. Cell lysates were analyzed by immunoblotting.

**Extended Data Figure 2.**
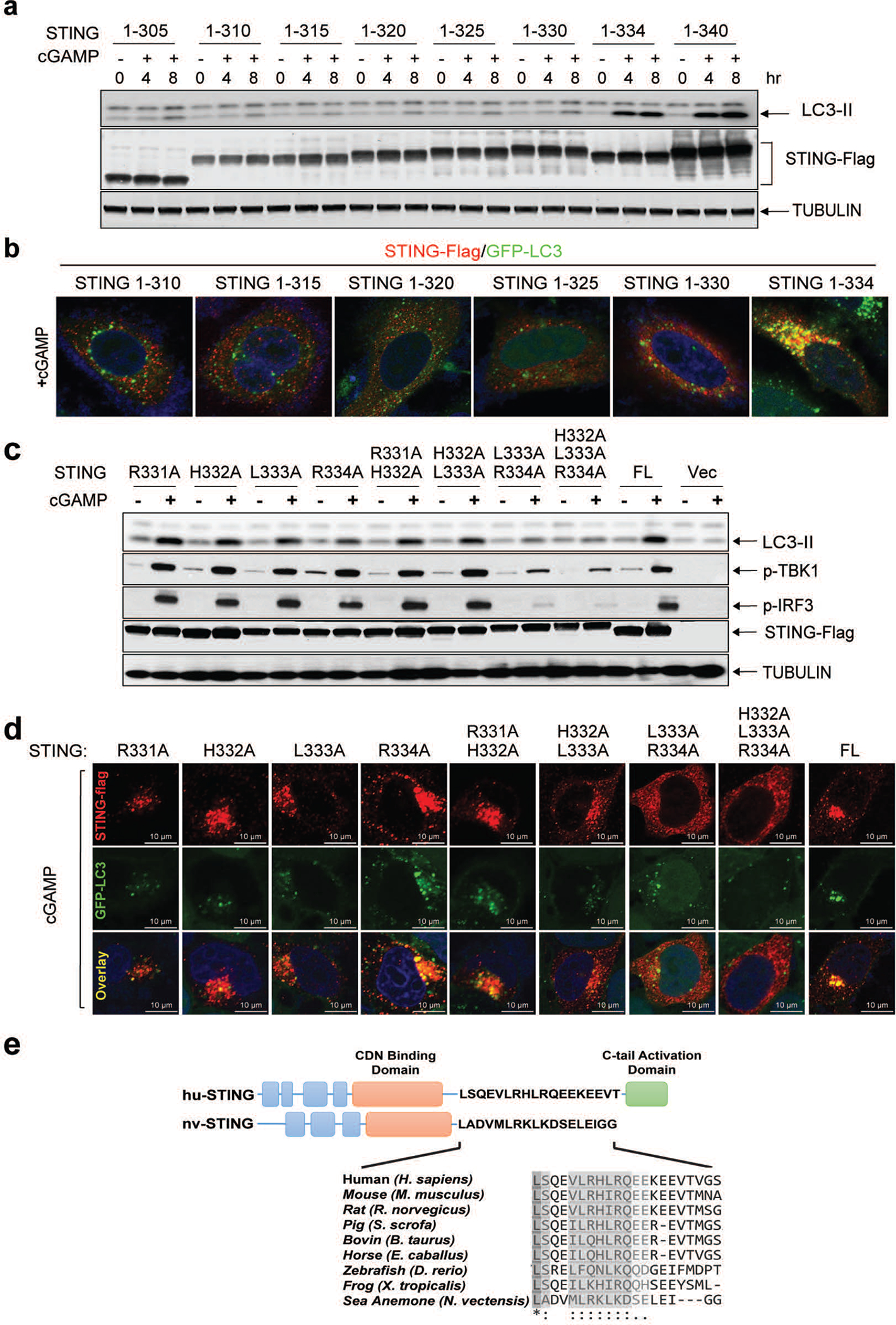
Delineation of the STING region (residues 330–334) required for LC3 lipidation. **a,** Expression plasmids encoding truncated STING mutants were transiently transfected into HEK293T cells for 24 hr, followed by stimulation with cGAMP for 4 hr. Cell lysates were analyzed by immunoblotting. **b,** Expression plasmids of indicated STING truncation mutants were transfected into HeLa GFP-LC3 cells for 24 hr. The cells were stimulated with cGAMP followed by immunostaining and fluorescence microscopy. **c&d,** L333 and R334 of STING are important for LC3 lipidation and TBK1 activation. **c,** Expression plasmids encoding full-length STING harboring the indicated mutations were transiently transfected into HEK293T cells, followed by stimulation with cGAMP. Cell lysates were analyzed by immunoblotting. FL: full-length. **d,** Indicated STING mutants were transfected and stimulated as described in **c** followed by immunostaining and fluorescence microscopy. **e,** Schematic of functional domains and residues of human (huSTING) and sea anemone STING (nvSTING), highlighting the evolutionary conservation of the CDN binding domain but not the C-terminal activation domain.

**Extended Data Figure 3.**
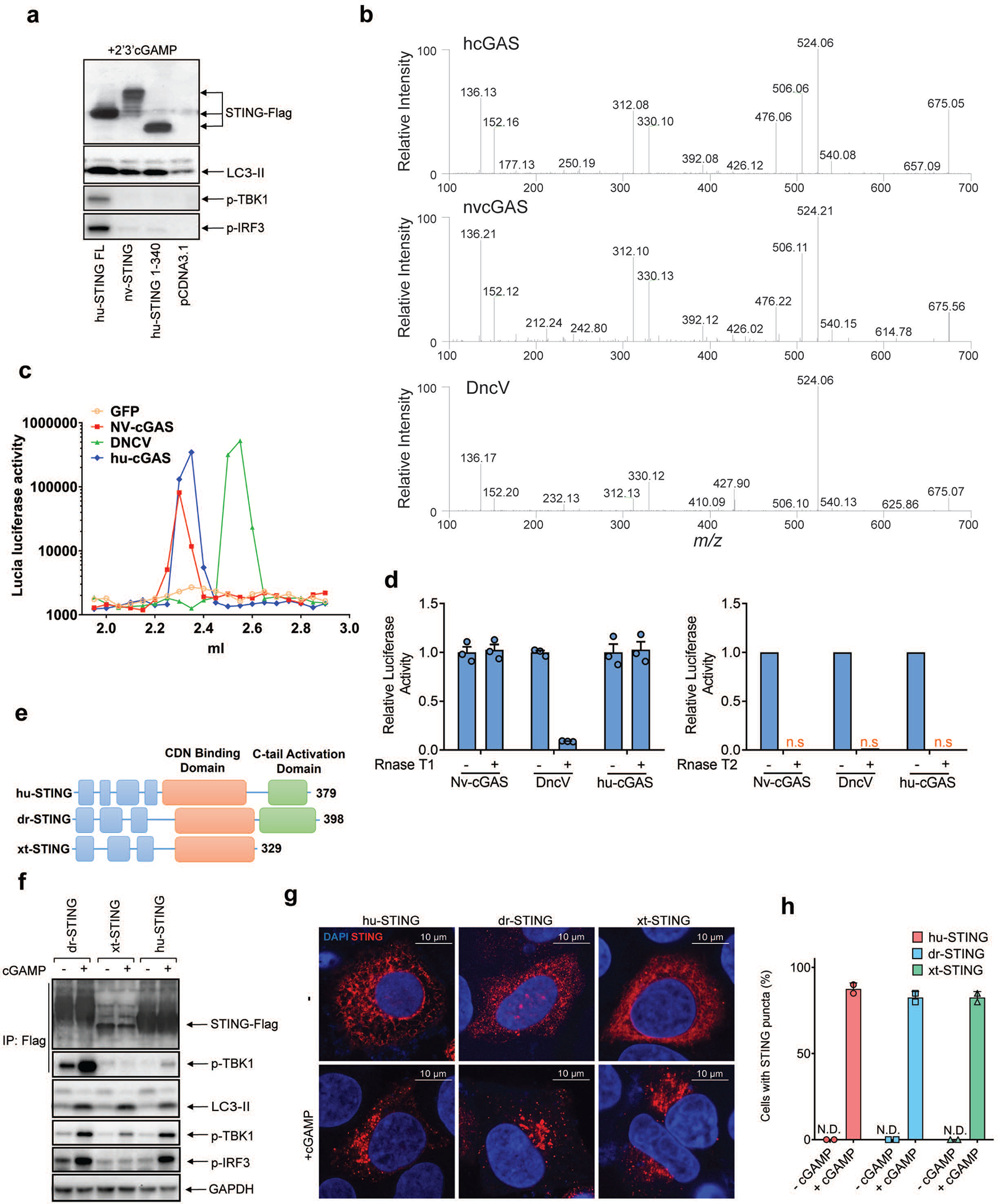
STING-induced LC3 conversion is a primordial function of the cGAS-STING pathway. **a,** Sea anemone STING (nvSTING) induces LC3 conversion but not TBK1 activation in response to 2’3’-cGAMP. HEK293T cells were transfected with expression plasmids encoding full-length (FL) human STING (huSTING), huSTING (1–340), or nvSTING for 24 hr and then treated with 2’3’-cGAMP for 3 hr. Cell lysates were analyzed by immunoblotting. **b**-**d,** Sea anemone cGAS (nvcGAS) produces 2’3’-cGAMP. **b,** Expression vectors encoding human cGAS (hucGAS), nvcGAS, or DncV were transfected into HEK293T cells for 24 hr. Small molecules were extracted from cells for analysis of cGAMP isomers by tandem mass spectrometry. Mass spectra of cGAMP from nvcGAS-expressing cells match those from hucGAS-expressing cells but not those from DncV-expressing cells. **c,** Similar to **b** except that the small molecule extracts were fractionated using a monoQ column, and each fraction was delivered into THP1-ISG luciferase reporter cells for measuring the CDN activity. **d,** CDNs produced by hucGAS and nvcGAS, but not that by DncV, is resistant to digestion by RNase T1. Small molecule extracts from HEK293T cells expressing the indicated CDN synthases were treated with RNase T1 or RNaseT2 or left untreated before delivery into THP1-ISG luciferase reporter cells to measure the CDN activity. Mean±SEM was shown (n=3) in the group treated with RNase T1. **e,** Domain organization of STING from human (hu), Danio rerio (dr), and Xenopus tropicalis (xt). **f,** Xenopus STING stimulates LC3 lipidation but not IRF3 phosphorylation. HEK293T cells were transfected with expression plasmids for STING-Flag from human, Danio, or Xenopus for 24 hr and then stimulated with cGAMP for 1 hr. A Flag antibody was used to immunoprecipitate STING from cell lysates followed by immunoblotting with the indicated antibodies. **g,** cGAMP stimulates formation of perinuclear puncta of STING from different species. Hela cells transiently expressing human, Danio, or Xenopus STING-Flag were stimulated with cGAMP for 3 hr. Cells were immunostained with a Flag antibody followed by fluorescence microscopy. **h,** Quantification of the percentage of cells with STING peri-nuclear foci formation. The percentage of cells with STING foci formation was quantified from 100 cells (n = 2). All results in this figure are representative of at least two independent experiments. N.D., not detectable.

**Extended Data Figure 4.**
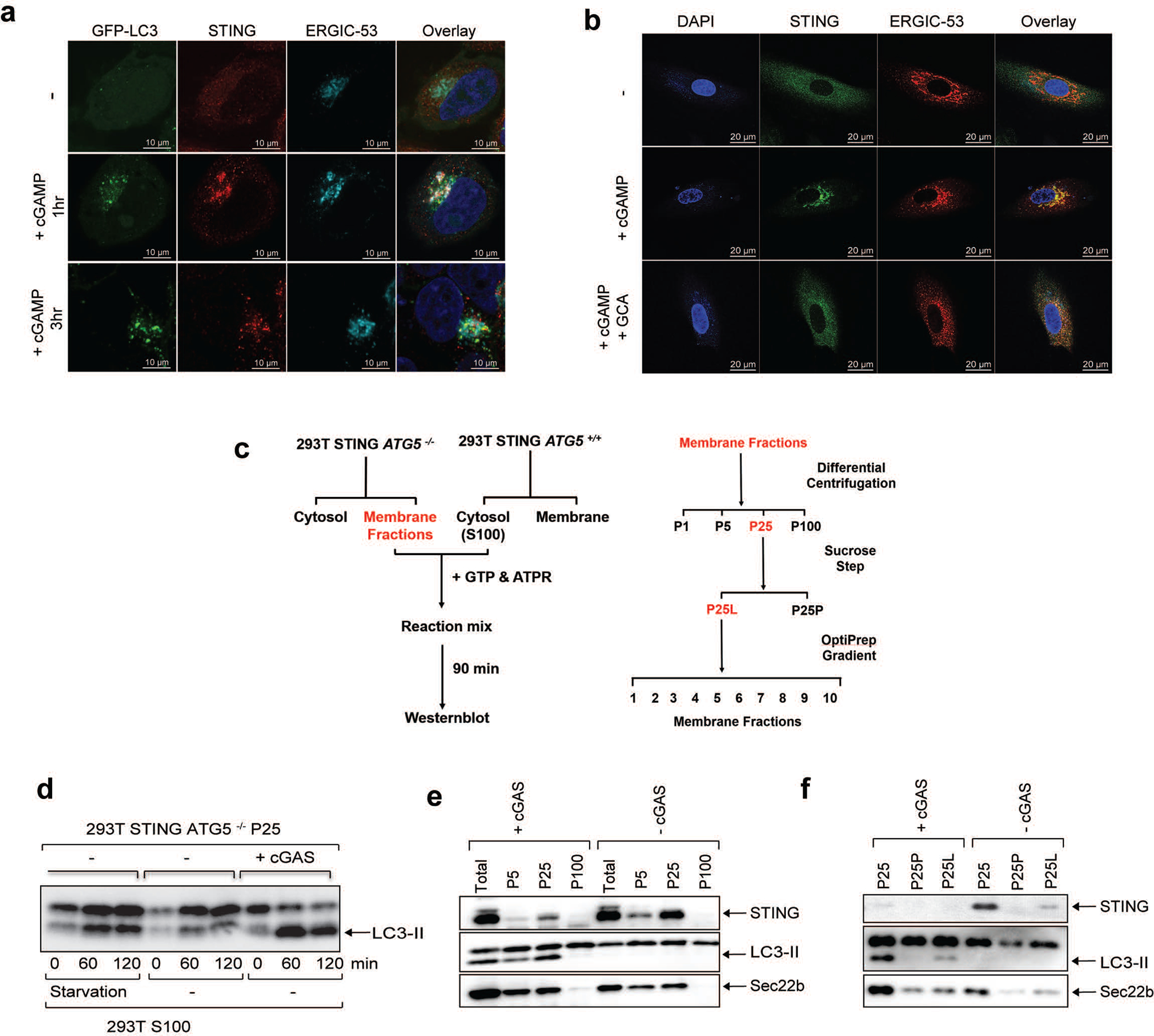
cGAMP stimulates STING translocation to ERGIC vesicles that promote LC3 lipidation. **a,** STING co-localizes with the ERGIC and autophagosomes in response to cGAMP stimulation. HeLa cells stably expressing GFP-LC3 and STING-Flag were stimulated with cGAMP for the indicated time followed by immunofluorescence microscopy. **b,** STING trafficking to the ERGIC is blocked by GCA. BJ cells were stimulated with cGAMP for 3 hr in the presence or absence of GCA. Cells were stained with DAPI or the indicated antibodies and examined by confocal microscopy. **c,** Procedures for in vitro reconstitution of cGAMP-induced LC3 lipidation and membrane fractionation. **d,** ATG5^−/−^ 293T cells stably expressing STING-Flag were transfected with a cGAS expression plasmid or an empty vector for 24 hr. Membrane pelleted at 25,000 g (P25) from these cells was incubated with cytosolic extracts (S100) from starved or untreated 293T cells in the presence of GTP and an ATP regenerating (ATPR) system. After incubation at 30^o^C for 90 min, the reaction mixtures were analyzed by immunoblotting. **e,** Similar to **c** except that different organelle membranes enriched by differential centrifugation were prepared and incubated with cytosol (S100) from HEK293T cells to detect LC3 lipidation. **f,** Similar to **c** except that P25 membranes were further fractionated by sucrose ultracentrifugation to generate P25P (pellet) and P25L (light) and incubated with cytosol (S100) from HEK293T cells to detect LC3 lipidation.

**Extended Data Figure 5.**
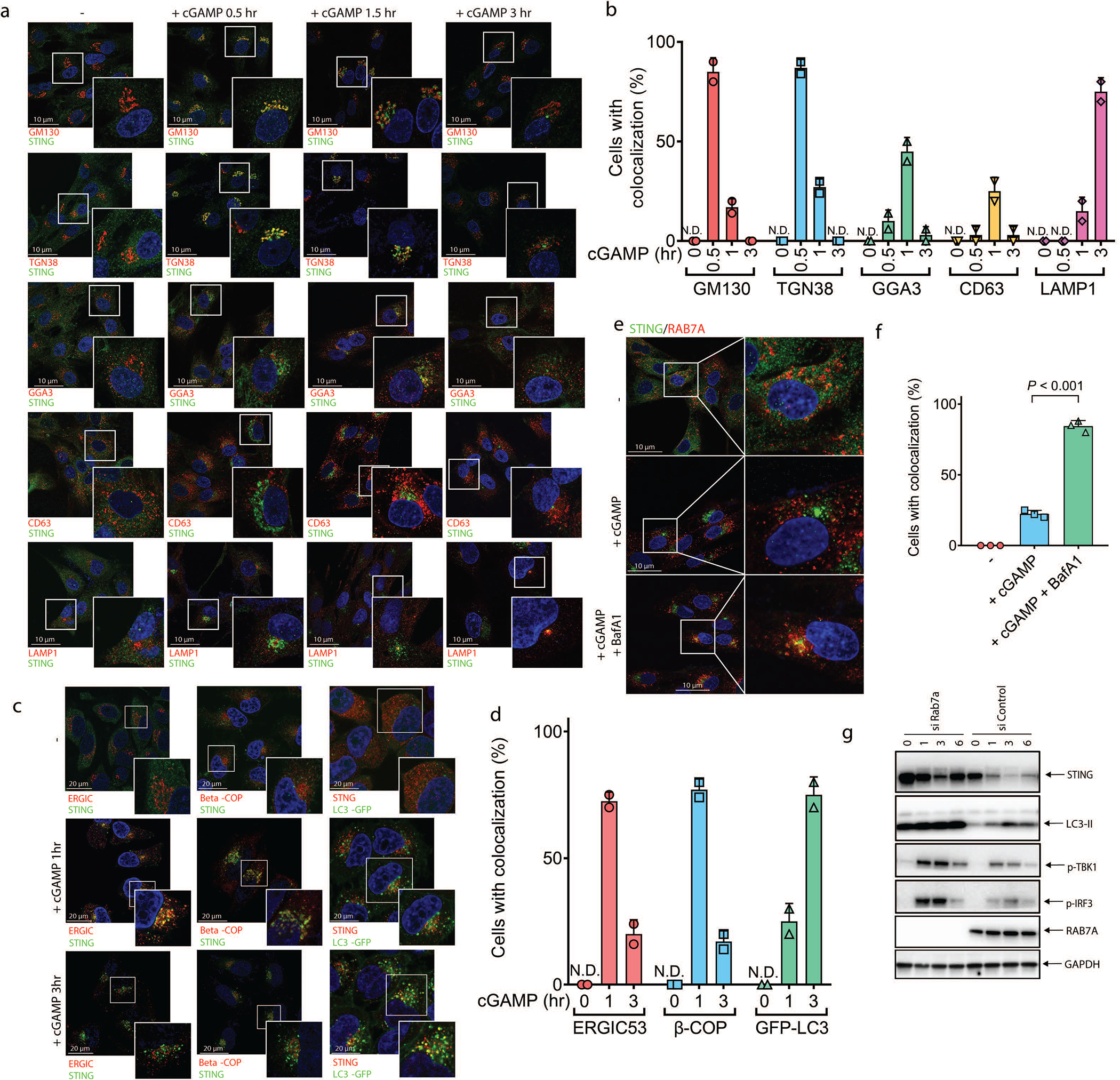
cGAMP-bound STING traffics through the Golgi and endosomes or the ERGIC and autophagosomes before reaching lysosomes. **a,** BJ cells were stimulated with cGAMP for the indicated time. Cells were immunostained with a STING antibody together with an antibody against GM130 (cis-Golgi), TGN38 (trans-Golgi), GGA3 (post-Golgi vesicles), CD63 (late endosomes), or LAMP1 (lysosomes) followed by immunofluorescence microscopy. **b,** Quantification of the percentage of cells in which STING colocalized with different organelle markers in **a**. N.D., not detectable. The percentage was quantified from 50 cells (n = 2). All results in this figure are representative of at least two independent experiments. **c,** cGAMP induces trafficking of STING to ERGIC, COP-I vesicles, and LC3 autophagosomes. HeLa cells stably expressing STING-Flag and GFP-LC3 were stimulated with cGAMP for the indicated time. Cells were immunostained with antibodies specific for Flag (to detect STING), ERGIC53 (ERGIC) or beta-COP (COP1 vesicles) followed by fluorescence microscopy. **d,** Quantification of the percentage of cells in which STING colocalized with different organelle markers in **c**. N.D., not detectable. The percentage was quantified from 50 cells (n = 2). **e,** BJ cells were stimulated with cGAMP in the presence or absence of Bafilomycin A1 (BafA1). Cells were immunostained with an antibody specific for STING or RAB7A followed by microscopy. **f,** Quantification of the percentage of cells in which STING colocalized with RAB7A in **e**. N.D., not detectable. The percentage was quantified from 50 cells (n = 3, mean ±SD, two-tailed Student’s *t*-test). **g,** BJ cells were transfected with an siRNA targeting RAB7A or a control siRNA for 3 days before stimulation with cGAMP for indicated time. Cell lysates were analyzed by immunoblotting.

**Extended Data Figure 6.**
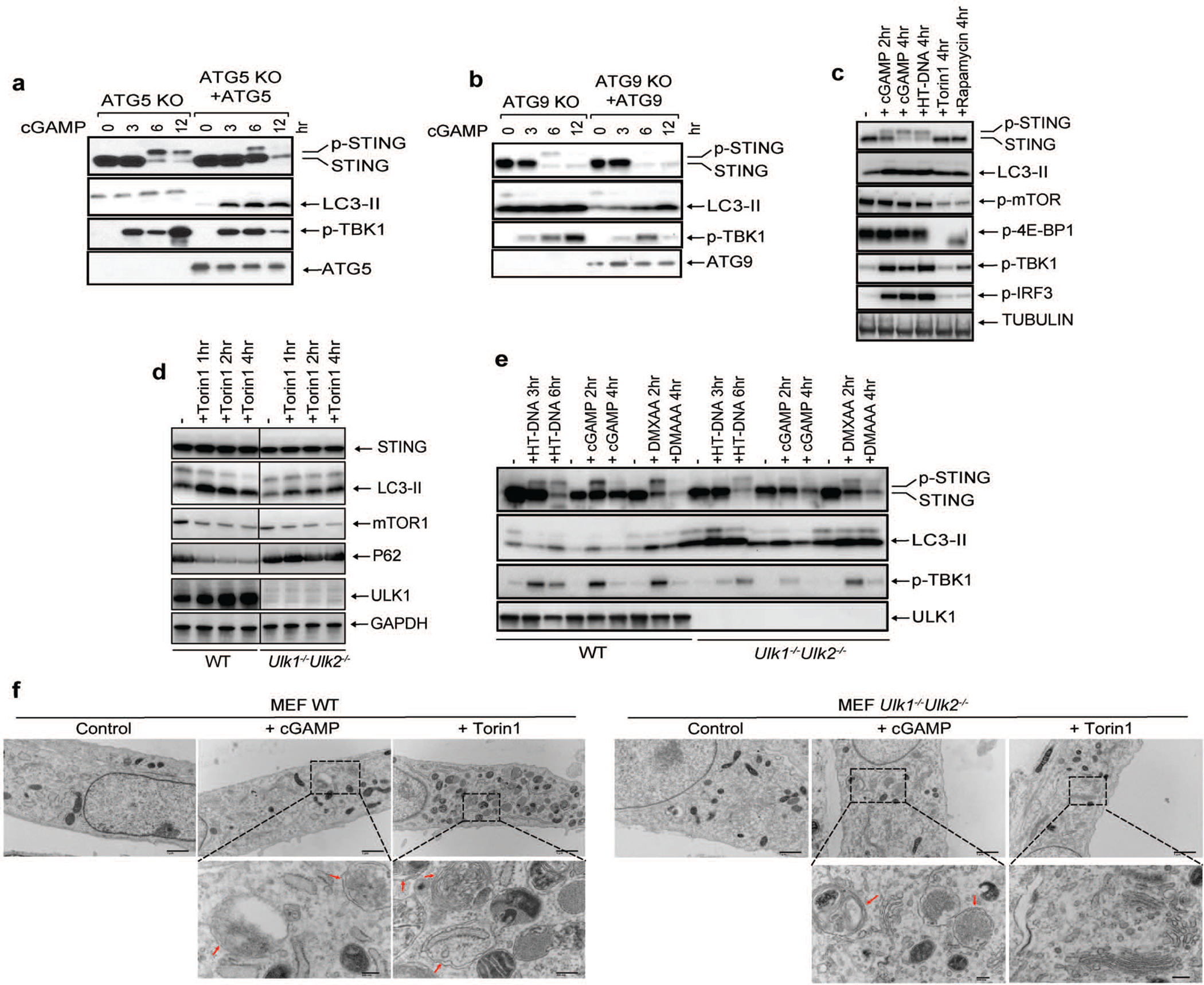
ULK kinases are dispensable for cGAMP-induced LC3 conversion and autophagosome formation. **a,** ATG5 is required for LC3 lipidation but dispensable for STING degradation induced by cGAMP. ATG5^−/−^ or ATG5-reconstituted BJ cells were stimulated with cGAMP for the indicated time followed by immunoblotting of cell lysates. **b,** ATG9 is dispensable for LC3 lipidation and STING degradation. Atg9^−/−^ or Atg9-reconstituted BJ cells were stimulated with cGAMP for the indicated time followed by immunoblotting of cell lysates. **c,** STING activation does not induce mTOR inhibition. BJ cells were treated with cGAMP, HT-DNA, Torin 1, or Rapamycin for the indicated time followed by immunoblotting of cell lysates. **d,** Loss of ULK1 and ULK2 impairs LC3 conversion and p62 degradation induced by mTOR inhibition.. Wild type and *Ulk1*^*−/−*^*Ulk2*^*−/−*^ MEF cells were treated with torin1 at the indicated time followed by immunoblotting of cell lysates. **e,** STING-induced LC3 conversion is independent of ULK1 and ULK2. Wild type and Ulk1−/−Ulk2−/− MEF cells were treated with cGAMP, HT-DNA, or DMXAA for the indicated times followed by immunoblotting of cell lysates. **f,** Electron micrographs of Wild type and *Ulk1*^*−/−*^*Ulk2*^*−/−*^ MEF cells stimulated with cGAMP or Torin1. Boxed areas are enlarged to show double-membrane organelles that represent autophagosomes. Red arrows highlight double-membrane characteristic of autophagosomes in stimulated cells. Scale bar is 1 μm for original picture and 200 nm for the zoomed in picture.

**Extended Data Figure 7.**
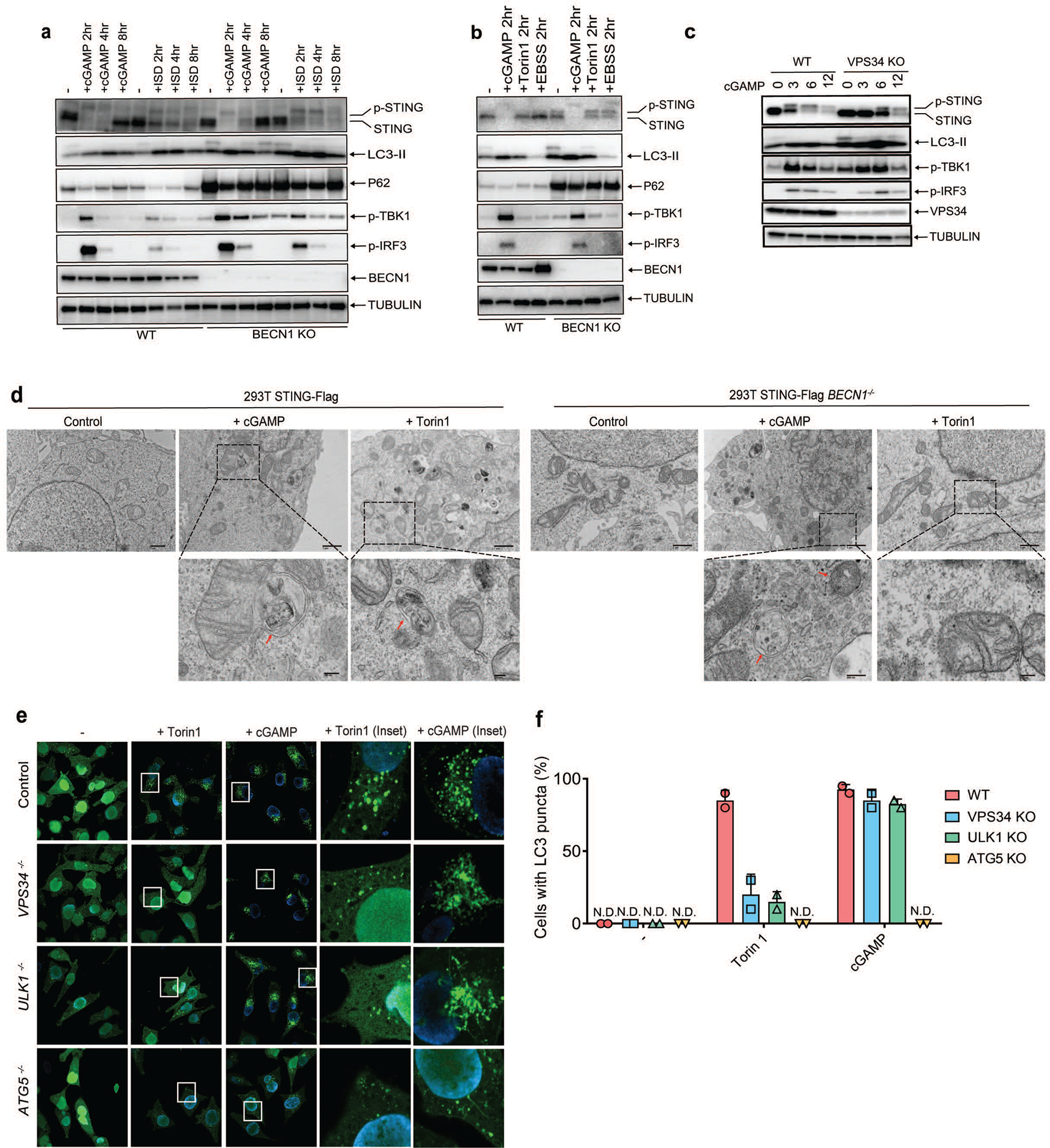
STING-induced LC3 conversion does not require Beclin-1 (BECN1) or VPS34. **a,** BECN1 is dispensable for LC3 conversion triggered by cGAMP. Wild type and *BECN1*^*−/−*^ BMDM were stimulated with cGAMP or HT-DNA at the indicated time followed by immunoblotting of cell lysates. **b,** BECN1 is not essential for LC3 conversion in conventional autophagy. Wild type and *BECN1*^*−/−*^ BMDM were stimulated with cGAMP or Torin 1 or cultured in EBSS starvation media at the indicated time followed by immunoblotting of cell lysates. **c,** VPS34 depletion delayed cGAMP-induced STING degradation but not LC3 lipidation. VPS34 knock-out BJ cells were treated with cGAMP for the indicated time followed by immunoblotting of cell lysates. **d,** Electron micrographs of 293T STING-Flag and 293T STING-Flag *BECN1*^*−/−*^ cells, stimulated with cGAMP or Torin1. Boxed areas are enlarged to show double-membrane organelles that represent autophagosomes. Red arrow highlights double-membrane characteristic of autophagosomes in stimulated cells. Scale bar is 1 μm for original picture and 200 nm for the zoomed pictures. **e&f,** ULK1 and VPS34 are essential for LC3 puncta formation induced by Torin 1 but not by cGAMP. *ULK1*^*−/−*^*, VPS34*^*−/−*^*, or ATG5*^*−/−*^ Hela GFP-LC3 cells were treated with Torin 1 or cGAMP for the indicated time. GFP-LC3 puncta formation was visualized by fluorescence microscopy (e) and the percentage of cells with GFP-LC3 peri-nuclear foci formation was quantified (f). N.D., not detectable. The percentage of cells with LC3-GFP puncta was quantified from 100 cells (n = 2).

**Extended Data Figure 8.**
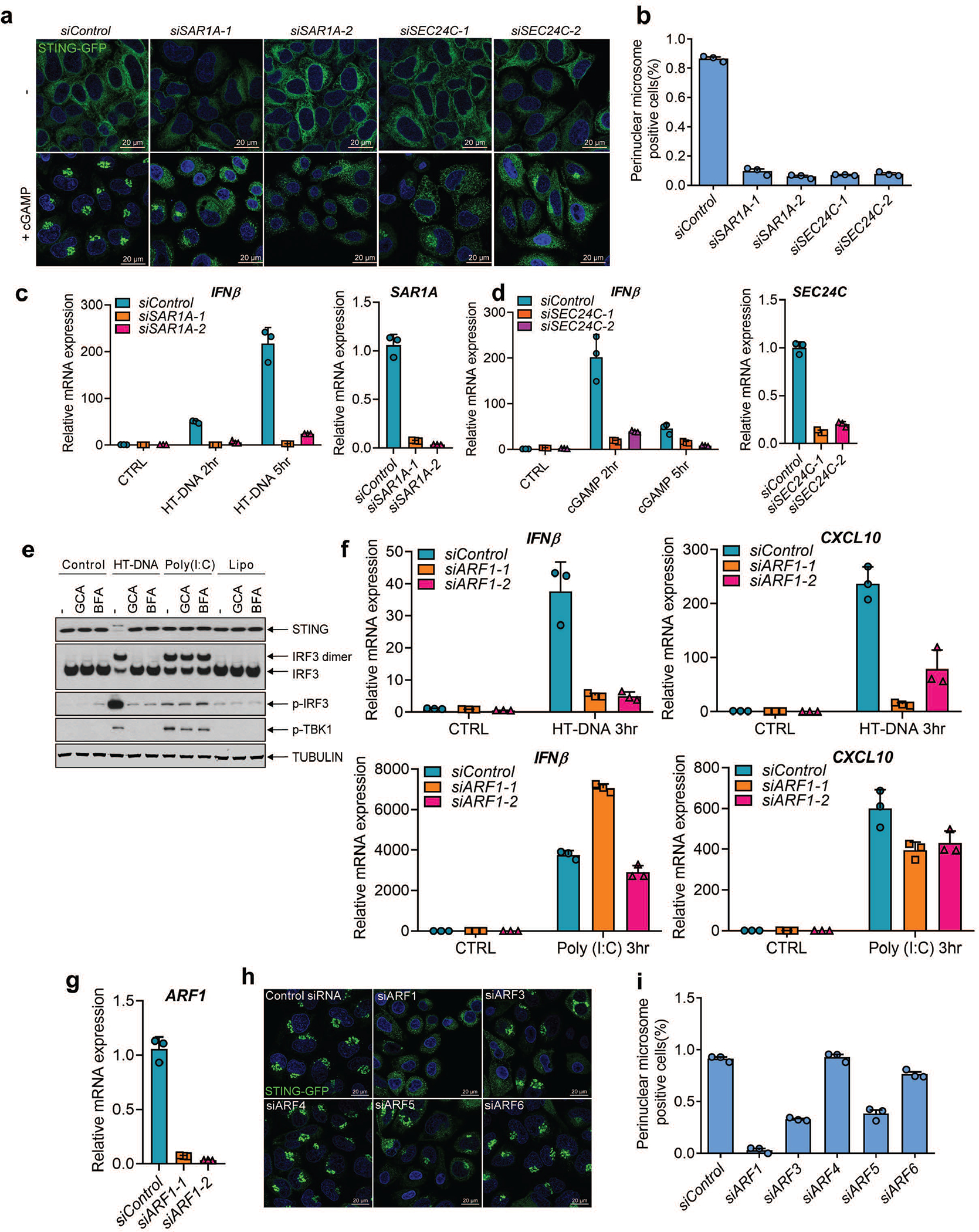
STING membrane trafficking and signaling requires SAR1A, SEC24C, and ARF GTPases. **a&b,** Hela STING-GFP cells were transfected with siRNAs targeting SAR1A, SEC24C, or luciferase (control) for 3 days before stimulation with cGAMP (75 nM) for 1 hr. STING-GFP puncta were detected by confocal microscopy (a) and quantified (b). The percentage of cells with STING-GFP puncta was quantified from three random fields (n = 3, mean ±SD).. **c,** BJ cells were transfected with siRNAs targeting SAR1A for 3 days before transfection with HT-DNA or Poly(I:C) for the indicated time. Total RNA was isolated to measure the expression of indicated genes by RT-qPCR. **d,** Similar to **c** except that Hela cells were transfected with siRNAs targeting SEC24C, and cells were stimulated with cGAMP or poly(I:C). Mean ± SD was shown. Data represent 2 independent experiments with 3 replicates. **e,** Membrane trafficking is essential for cytosolic DNA but not RNA signaling. BJ cells were stimulated with BFA or GCA before transfection with HT-DNA or poly (I:C) or Lipofectamine (Lipo) alone. Cell lysates were analyzed by native gel (for IRF3 dimerization) or SDS-PAGE followed by immunoblotting with the indicated antibodies. **f&g,** BJ cells were transfected with two different siRNAs targeting ARF1 before transfection with HT-DNA or Poly (I:C) for the indicated time. Total RNA was isolated to measure the expression of the indicated genes by RT-qPCR. Mean ± SD was shown. Data represent 2 independent experiments with 3 replicates. **h&i,** Hela STING-GFP cells were transfected with siRNAs targeting different ARF family members for 3 days and then stimulated with cGAMP (75 nM) for 1 hr. STING-GFP foci were detected by confocal microscopy (h) and quantified (i). The percentage of cells with STING-GFP puncta was quantified from three random fields (n = 3, mean ±SD).

**Extended Data Figure 9.**
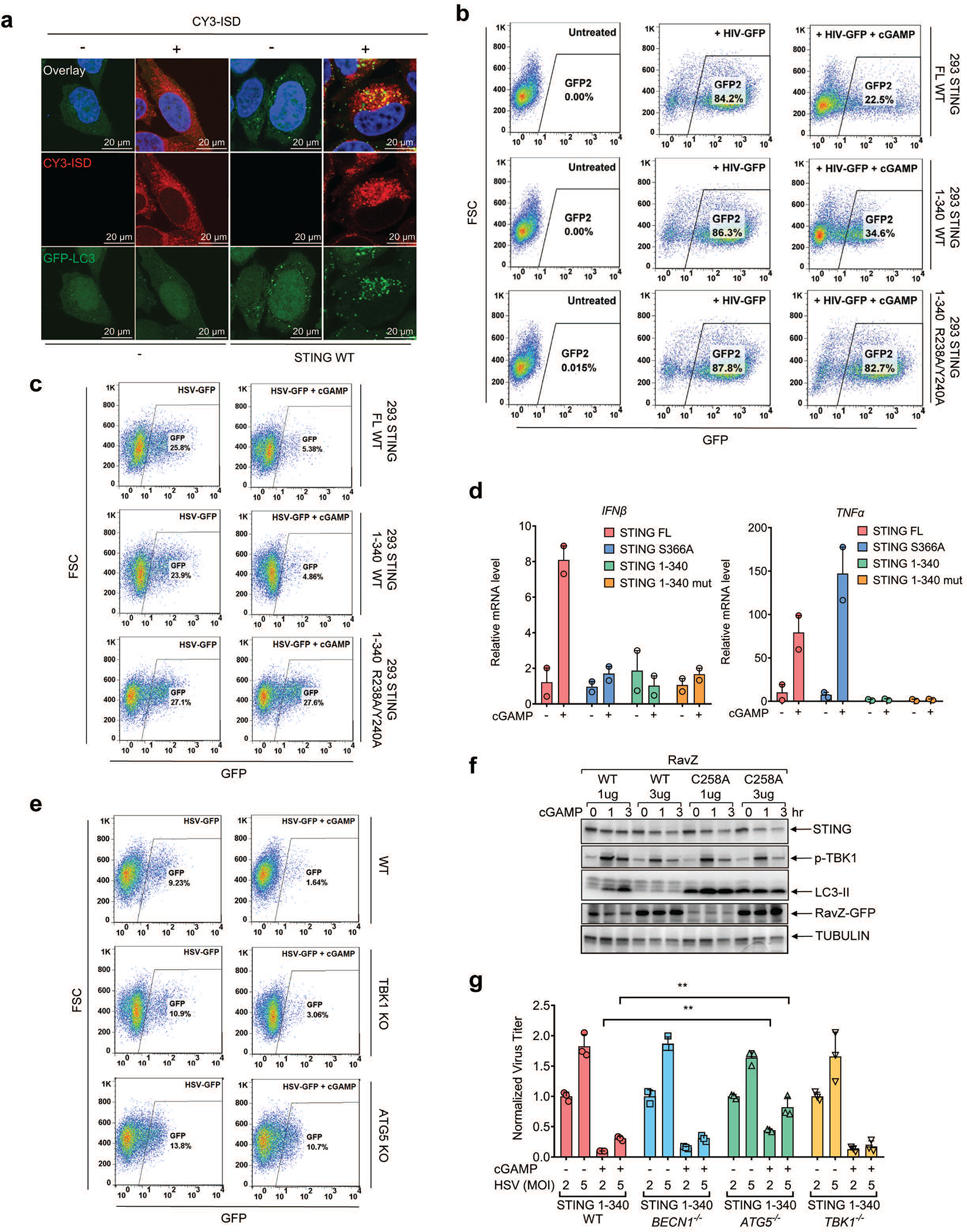
cGAMP induces anti-viral defense through autophagy. **a,** Cytosolic DNA colocalizes with LC3 vesicles in STING-expressing cells. CY3-ISD DNA was delivered into HeLa-GFP-LC3 cells (lacking endogenous STING) or those stably expressing STING in the presence of PFO, followed by fluorescence microscopy. Single cell images are shown, representing > 90 % of the cells under examination. **b&c,** cGAMP-induced activation of STING 1–340 enhances clearance of HIV-1 and HSV-1. 293T cells reconstituted with WT or mutant STING were stimulated with cGAMP and then infected with the pseudotyped HIV1-GFP virus for 24 hr (b) or HSV1-GFP for 18 hr (c). GFP^+^ cells were analyzed by FACS. The results are representative of two independent experiments. **d,** STING 1–340 does not induce IFN-β or TNFα. HEK293T cells stably expressing full length STING, STING (S336A), STING (1–340) or STING (1–340, R238A) were stimulated with cGAMP (2 μM) for 8 hr and mRNA was extracted for RT-qPCR analysis of IFN-β or TNFα gene expression. Representative data was shown from two independent experiments. n=2. Data are presented as mean ± SD. **e,** RavZ catalyzes LC3 deconjugation. 293T-STING stable cells were transfected with RavZ expression plasmids (WT or C258A mutant) for 36 hr and then stimulated with cGAMP for the indicated time. Cell lysates were analyzed by immunoblotting with the indicated antibodies. **f,** ATG5 knockout partially reverses cGAMP-mediated repression of HSV-1. ATG5 or TBK1 were knocked out using CRISPR in STING-expressing HEK293T cells. The cells were then infected with HSV-GFP with or without cGAMP treatment. FACS was performed to quantify relative virus GFP intensity in each cell line. **g,** ATG5 deficiency partially abrogated cGAMP-mediated suppression of HSV-1. BECN1, ATG5, or TBK1 was knocked out using CRISPR in STING-expressing HEK293T cells. The cells were then infected with HSV-1 ΔICP34.5 with or without cGAMP stimulation. qPCR using HSV-1 primers was performed to quantify relative viral genome equivalent (VGE) in each cell line. n=3. Data are presented as mean ± SD. n.s., not significant (two-tailed Student’s *t*-test).

**Extended Data Figure 10.**
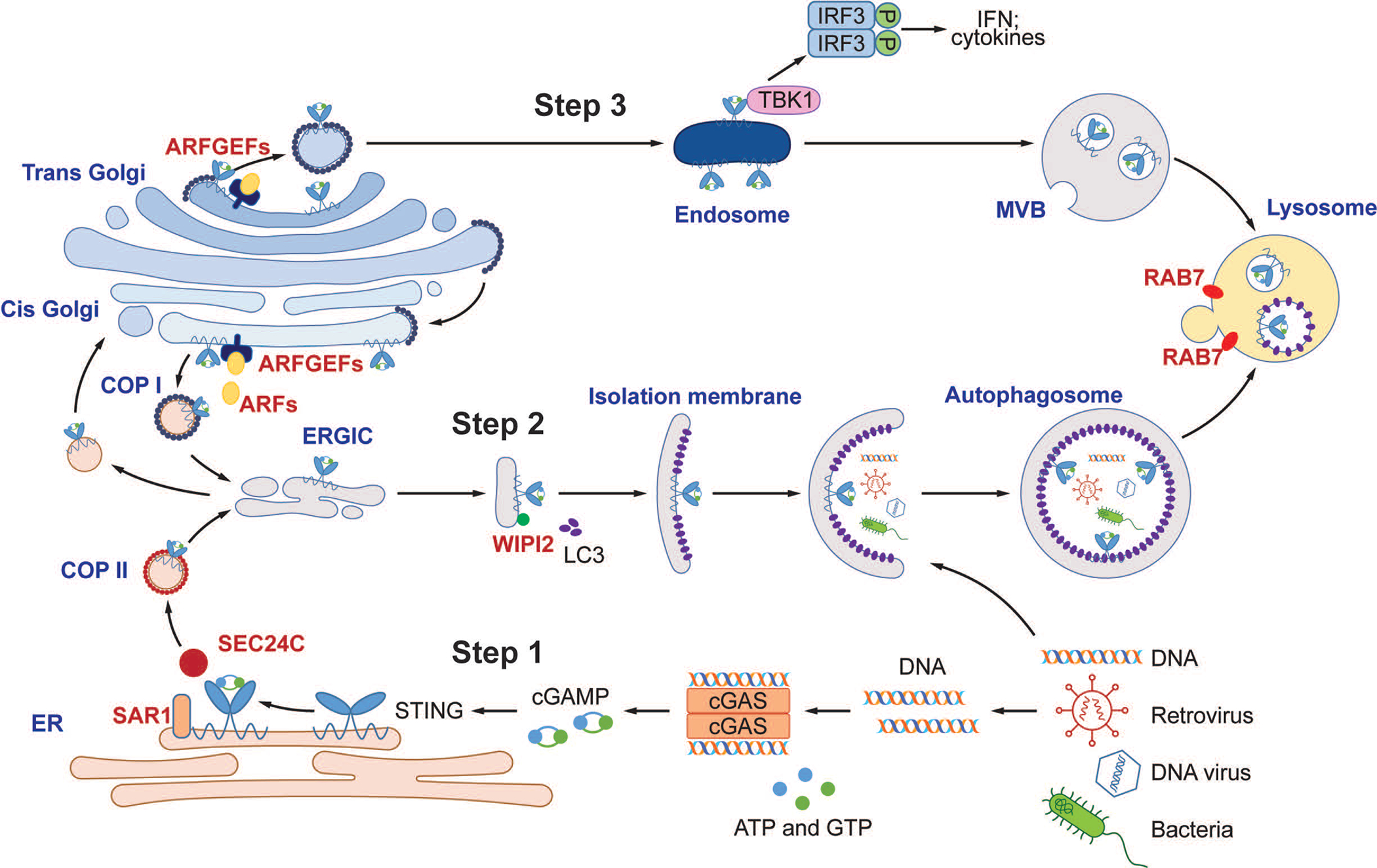
A model of DNA-induced autophagy and signaling through the cGAS-STING pathway. Step 1: DNA from pathogens or damaged cells activates cGAS to synthesize cGAMP. cGAMP binds to STING and triggers STING translocation from the ER to the ERGIC and Golgi in a process that depends on SAR1, SEC24C, and ARF family members. Step 2: The ERGIC, which contains cGAMP-bound STING, serves as a membrane source for LC3 recruitment and lipidation through a WIPI2-dependent mechanism. LC3-positive membranes target DNA and pathogens to autophagosomes, which are subsequently fused with lysosomes. Step 3: cGAMP-bound STING can also translocate through the trans-Golgi network (TGN) and endosomes to lysosomes for degradation via the multivesicular body (MVB) pathway. Both the MVB and autophagosome fuse with lysosomes in a process that requires RAB7 GTPase.

## Supplementary Material

Supplementary Video 1

Supplementary Video 2

Supplementary Video 3

Supplementary Video 4

Supplementary Video 5

Supplementary Video 6

Supplementary Information including tables

## Figures and Tables

**Figure 1.**
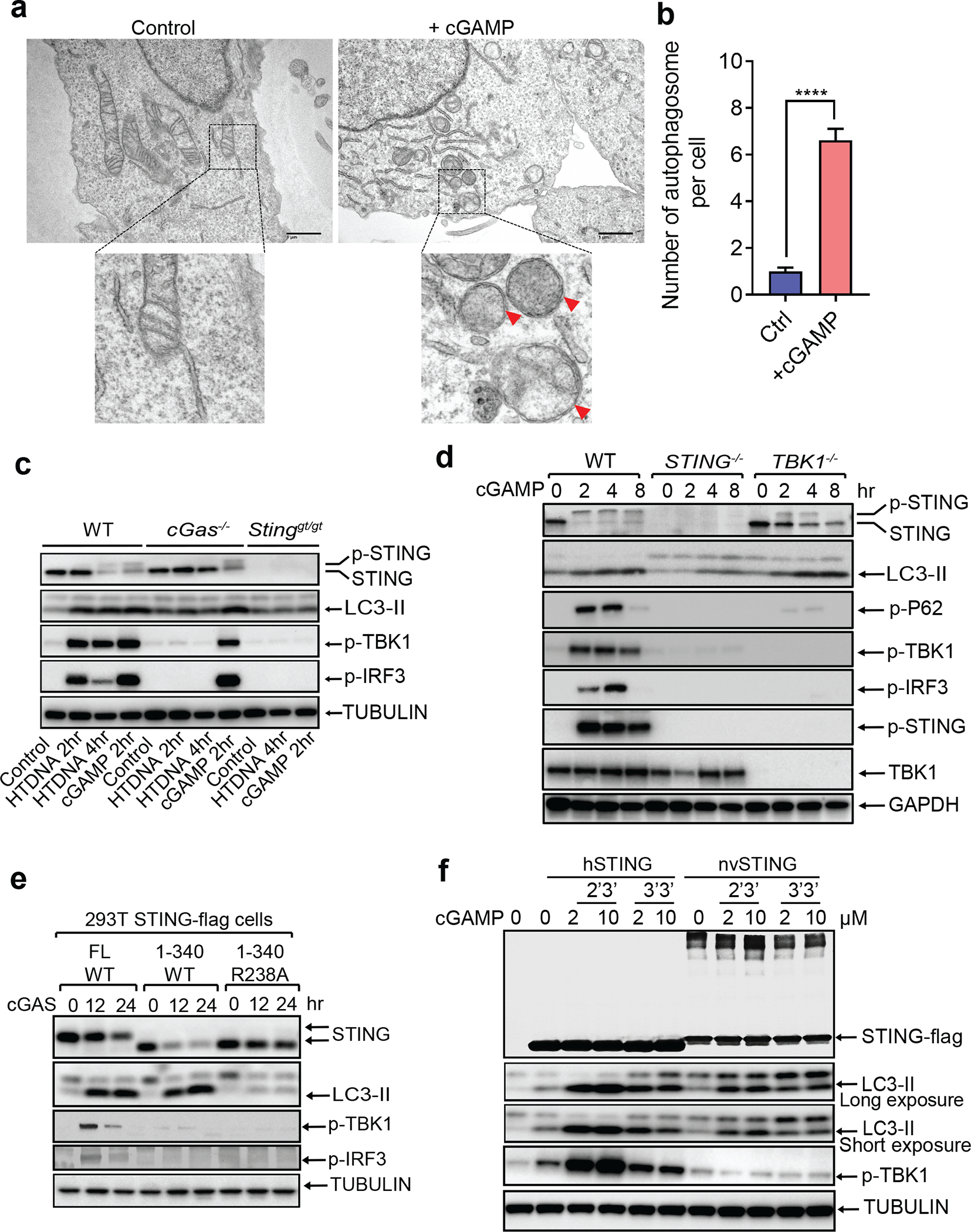
Autophagy induction by STING is evolutionarily conserved and separable from interferon induction. **a,** cGAMP induces autophagosome formation. Electron micrographs of BJ cells stimulated with cGAMP. Boxed areas are enlarged to show double-membrane organelles representing autophagosomes, as indicated by red arrowheads. **b,** Quantification of the number of double-membrane autophagosomes per cell was obtained by counting BJ cells stimulated with (n=32) or without cGAMP (n=39). Mean ±SEM are shown. **** p < 0.0001 (two-tailed Student’s *t*-test). **c,** DNA-induced LC3 lipidation requires cGAS and STING. WT, *cGas*^*−/−*^, or *Sting*^*gt/gt*^ primary MEF cells were stimulated with cGAMP or transfected with HT-DNA for the indicated time, followed by immunoblotting. **d,** TBK1 is dispensable for LC3 conversion. WT, *STING*^*−/−*^, or *TBK1*^*−/−*^ BJ cells were stimulated with cGAMP before cell lysates were analyzed by immunoblotting. **e,** HEK293T cells stably expressing the indicated STING proteins were transfected with a cGAS expression plasmid before cell lysates were analyzed by immunoblotting. **f,** nvSTING stimulates LC3 conversion but not TBK1 activation. HEK293T cells expressing huSTING or nvSTING were treated with indicated concentrations of 2’3’-cGAMP or 3’3’-cGAMP followed by immunoblotting.

**Figure 2.**
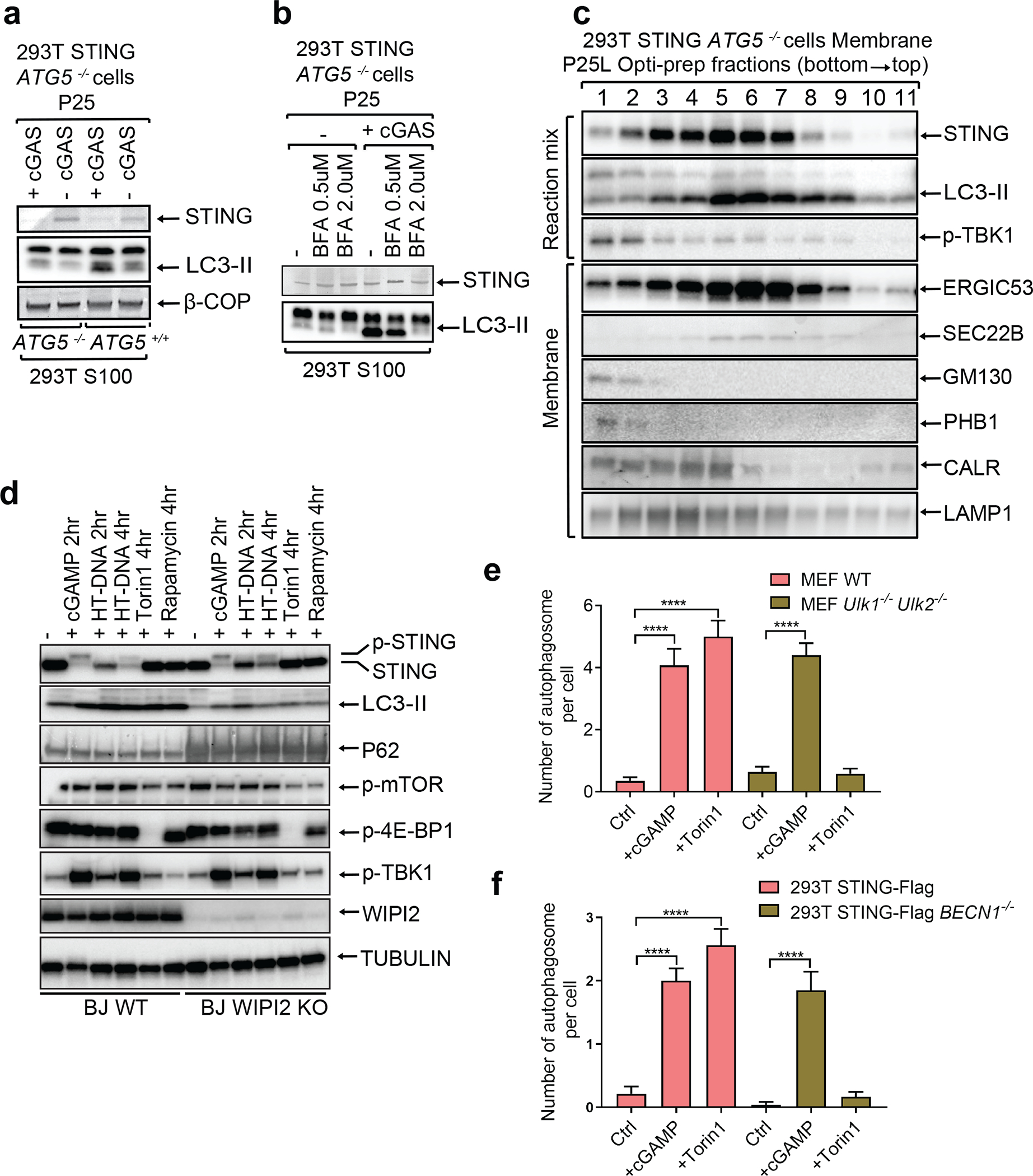
STING translocates to the ERGIC to trigger autophagosome formation. **a,** LC3 lipidation in vitro requires membranes from cGAS-stimulated cells and cytosolic extracts containing ATG5. *ATG5*^*−/−*^ HEK293T cells stably expressing STING were transfected with a cGAS expression plasmid for 18 hr before membrane pellets (P25) were prepared by differential centrifugation. The membranes were incubated with cytosolic extracts (S100) from HEK293T cells followed by immunoblotting analysis. **b,** Membrane trafficking of STING is important for LC3 lipidation in vitro. Similar to **a** except that BFA was added to 293T-STING *ATG5*^*−/−*^ cells at indicated concentrations before cells were transfected with a cGAS expression plasmid. **c,** ERGIC fractions are enriched with LC3 lipidation activity. Similar to **a** except that P25 membranes were further fractionated by Opti-Prep gradient ultracentrifugation. The reaction mixtures and each fraction from the ultracentrifugation were analyzed by immunoblotting with the indicated antibodies. **d,** WIPI2 is required for STING-induced LC3 conversion. WIPI2 knock-out BJ cells were treated with cGAMP, HT-DNA, Torin 1, or rapamycin for the indicated time followed by immunoblotting of cell lysates. **e&f,** cGAMP induces autophagosome formation independently of ULK1 and BECN1. WT and *Ulk1*^*−/−*^*Ulk2*^*−/−*^ MEF cells **(e)**, and 293T STING-Flag and 293T STING-Flag *BECN1*^*−/−*^ cells **(f)** were stimulated with cGAMP or Torin 1 as indicated. Quantification was shown as the number of double-membrane autophagosomes per cell by counting in MEF cells (n=17, 13, 13, 25, 20, 29; left to right) or 293T STING cells (n=14, 15, 16, 23, 20, 24; left to right). Mean±SEM are shown. **** p < 0.0001 (two-tailed Student’s *t*-test).

**Figure 3.**
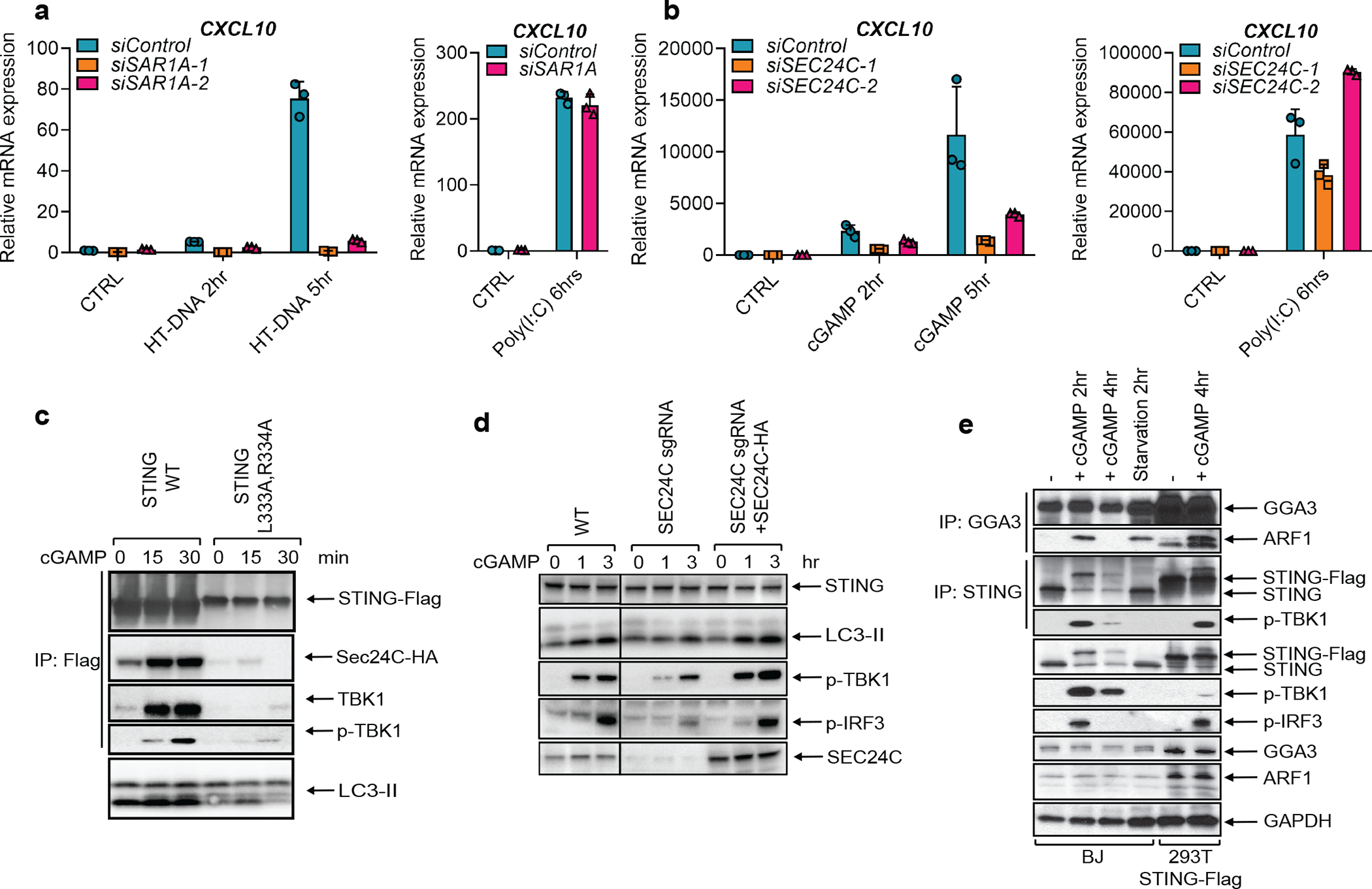
SAR1A and SEC24C are essential for STING trafficking and signaling. **a,** BJ cells were transfected with siRNAs targeting SAR1A for 3 days before transfection with HT-DNA or Poly(I:C) for the indicated time. Total RNA was isolated to measure the expression of indicated genes by RT-qPCR. **b,** Similar to **a** except Hela cells were transfected with siRNAs targeting SEC24C, and cells were stimulated with cGAMP or poly(I:C). Mean ± SD was shown. Data represent 2 independent experiments with 3 replicates **c,** cGAMP induces STING binding to SEC24C. HEK293T cells stably expressing SEC24C-HA and WT STING-FLAG or the indicated STING mutant were stimulated with cGAMP before cell lysates were prepared for immunoprecipitation using Flag antibody. Precipitated proteins were analyzed by immunoblotting. **d,** SEC24C is required for LC3 lipidation and IRF3 phosphorylation. HEK293T cells stably expressing STING were infected with lentiviruses harboring SEC24C sgRNA to deplete endogenous SEC24C. To restore SEC24C expression, an aliquot of the cells was infected with lentiviruses expressing sgRNA-resistant SEC24C cDNA. The cells were stimulated with cGAMP followed by immunoblotting. **e,** cGAMP stimulates ARF1 GTPase activity. BJ cells were treated with cGAMP or starved for the indicated time before cell lysates were immunoprecipitated with an antibody against GGA3 or STING, followed by immunoblotting with the indicated antibodies.

**Figure 4.**
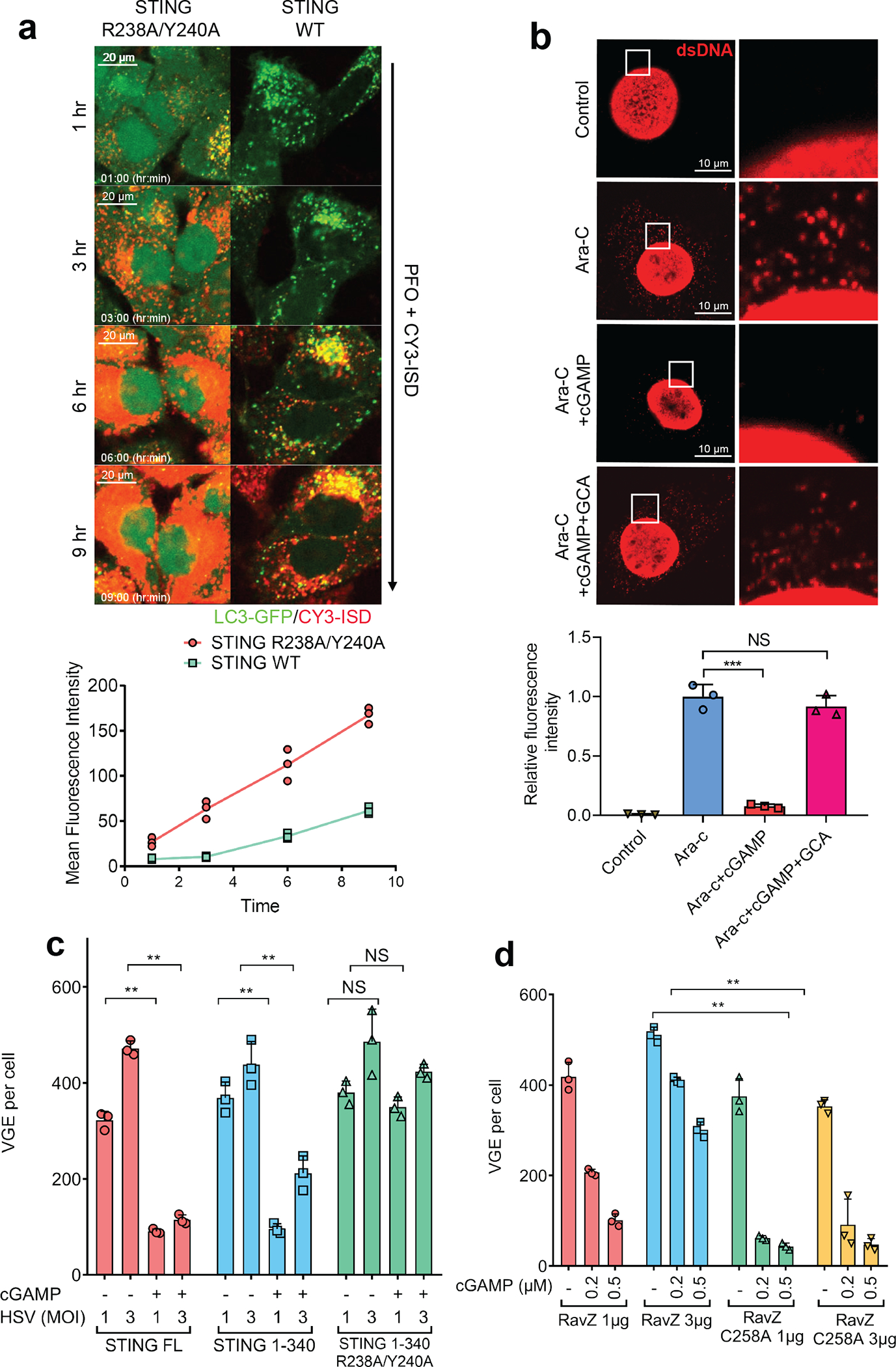
cGAMP-induced autophagy mediates the clearance of cytosolic DNA and DNA viruses. **a,** cGAMP binding by STING enhances the clearance of cytosolic DNA. Cy3-ISD was delivered into HeLa-GFP-LC3 cells stably expressing WT or mutant (R238A/Y240A) STING. Live cell imaging was carried out with still frames shown at the indicated times. Each mean fluorescence intensity of Cy3-ISD was calculated using Image J from three different areas, each of which contains three cells (bottom graph). n=3. **b,** cGAMP enhances degradation of cytosolic DNA generated by DNA damage. MEF cells were treated with Ara-C for 12 hr and then stimulated with cGAMP in the presence or absence of golgicide A (GCA) for another 12 hr. Cytosolic DNA was stained with a dsDNA-specific antibody. Intensity of cytosolic DNA staining was determined using Image J by deducting nuclear staining. Calculations were based on five cells from three different areas (bottom graph). Data was shown as mean± SEM. *** p < 0.001 (n=3, two-tailed Student’s *t*-test); NS, not significant (significance level, α = 0.01). **c,** Autophagy induction through STING 1–340 is sufficient to suppress HSV-1 replication. HEK293T cells stably expressing WT or mutant STING were stimulated with cGAMP and then infected with HSV-1 ΔICP34.5 for 12 hr at a MOI of 1 or 3. Viral DNA in the infected cells was quantified by qPCR using primers targeting the HSV-1 genome. VGE: virus genome equivalent. Mean ± SD was shown. Data represent 2 independent experiments with 3 replicates. n.s., not significant. (n=3, two-tailed Student’s *t*-test). **d,** LC3 deconjugation by RavZ abrogates cGAMP’s anti-viral effects. HEK293T-STING stable cells transiently expressing WT or C258A RavZ were stimulated with indicated concentrations of cGAMP before infection by HSV-1 △ICP34.5 for 8 hr. Viral DNA in infected cells was measured by qPCR to calculate VGE. Data are presented as mean ± SD. (n=3, two-tailed Student’s *t*-test).
