# Supplementary material for: Autophagy Induction via STING Trafficking Is a Primordial Function of the cGAS Pathway
Source: Nature. Author manuscript; Available in PMC 2022 Aug 26. (PMC9417302; doi:10.1038/s41586-019-1006-9)
Supplement: Supplementary Information including tables [file NIHMS1829177-supplement-Supplementary_Information_including_tables.docx]

**Supplementary information (SI)**

SI includes Supplementary Results, Supplementary Tables and References

**Supplementary Results**

**STING vesicle trafficking but not TBK1 phosphorylation of STING is required for cGAMP-induced LC3 lipidation and STING degradation in the lysosome**

Inhibitors of TBK1 (BX785 and MRT 67307) or IKK (TPCA1) did not impair cGAMP-induced LC3 lipidation or STING degradation in BJ cells (Extended Data Fig. 1d, e). Similarly, TBK1-deficient BJ cells formed autophagosomes normally in response to cGAMP stimulation (Extended Data Fig. 1f). We have previously shown that TBK1 phosphorylation of Ser-366 of human STING is necessary to recruit IRF3 to STING and TBK1, permitting TBK1 to phosphorylate IRF3^1^. Consistent with a dispensable role for TBK1 in cGAMP-induced autophagy, STING (S366A) still mediated robust LC3 lipidation and was degraded in response to cGAS transfection (Extended Data Fig. 1g). A STING mutation that cause STING associated vasculopathy with onset in infancy (SAVI)^2^, V155M, triggered constitutive formation of STING and LC3 puncta in the absence of cGAMP (Extended Data Fig. 1h, i). Confocal immunofluorescence and live cell imaging revealed that bafilomycin A1 (BafA1), which inhibits lysosomal acidification, prolonged the appearance of STING and LC3 puncta induced by cGAMP; in contrast, brefeldin A (BFA), which inhibits ER-to-Golgi membrane trafficking, blocked the formation of these puncta (Extended Data Fig. 1j, k, and Supplementary Videos 2, 3). These results indicate that cGAMP binding, not S366 phosphorylation of STING by TBK1, is important for STING-induced autophagy and STING trafficking. BafA1 and chloroquine, but not the proteasome inhibitors MG132 and Velcade, inhibited cGAMP-induced degradation of STING (Extended Data Fig. 1l), indicating that STING is degraded in the lysosome but not the proteasome. BFA blocked cGAMP-induced phosphorylation of TBK1, STING and IRF3 as well as LC3 lipidation and STING degradation (Extended Data Fig. 1l), suggesting that STING trafficking is essential for all downstream signaling events.

**Sea Anemone cGAS produces 2’3’-cGAMP**

Our finding that 2’3’-cGAMP was more potent than 3’3’-cGAMP in stimulating nvSTING prompted us to test the phosphodiester linkages in the cGAMP product generated by nvcGAS. We transfected HEK293T cells with expression plasmids encoding hucGAS, nvcGAS, or dinucleotide cyclase in Vibro cholerae (DncV), which is known to produce 3’3’-cGAMP^3^. Small molecule extracts from the transfected cells were analyzed by tandem mass spectrometry, ion exchange chromatography (monoQ) and enzyme digestion (RNase T1 and T2), all of which showed that the cGAMP produced by nvcGAS was 2’3’-cGAMP but not 3’3-cGAMP (Extended Data Fig. 3b, 3c and 3d). These results demonstrate that nvcGAS produces 2’3’-cGAMP rather than 3’3’-cGAMP.

**STING traffics to lysosomes via two Rab7a-dependent routes**

Imaging at different time points revealed that STING colocalized with markers of several organelles and vesicles, specifically following two routes to the lysosome: route 1 - trans-Golgi network (TGN38), endosomes derived from TGN (GGA3), late endosome (CD63), lysosome (LAMP1) (Extended Data Fig. 5a, b); route 2 - ERGIC (ERGIC53), autophagosome (GFP-LC3) and lysosome (LAMP1) (Extended Data Fig. 5c, d). In the presence of BafA1, STING colocalized with Rab7a, a GTPase associated with the late endosome (Extended Data Fig. 5e, f). Knockdown of Rab7a by RNAi slowed STING degradation and led to accumulation of phosphorylated TBK1 and IRF3 (Extended Data Fig. 5g). Rab7a depletion also enhanced LC3 conversion even in the absence of stimulation (Extended Data Fig. 5g), consistent with an important role for Rab7a in the degradation of LC3-positive autophagosomes^4,5^.

**cGAMP-induced autophagy is independent of the Vps34/Beclin-1 complex**

We also tested if the VPS34/Beclin 1 (BECN1) complex, which can generate PI3P for WIPI2 recruitment^6-8^, is necessary for STING-mediated LC3 lipidation. Macrophages deficient in BECN1 (from Becn1 floxed/floxed, LysM-Cre mice) showed higher LC3 conversion than autophagy-sufficient (Becn1 floxed/floxed) cells after cGAMP delivery and HT-DNA transfection (Extended Data Fig. 7a). In the presence of Torin 1, the BECN1-deficient cells accumulated P62 but LC3 lipidation was not impaired (Extended Data Fig. 7b), which is consistent with other recent reports^9-11^. Electron microscopy experiments showed that deletion of BECN1 in 293T-STING cells abrogated double-membraned autophagosome formation induced by Torin 1 but not cGAMP (Fig. 2f and Extended Data Fig. 7d). Similarly, knockout of VPS34 in BJ cells did not abolish LC3 conversion but resulted in the additional accumulation of LC3-II and partial stabilization of STING after cGAMP treatment (Extended Data Fig. 7c). As PI3P can be generated independently of the VPS34/BECN1 complex^11^, further work is required to determine the PI3P source for STING-mediated lipidation. Detection of GFP-LC3 puncta in HeLa cells showed that deletion of ULK1 or VPS34 diminished the number of LC3 puncta induced by Torin 1 but not cGAMP (Extended Data Fig. 7e and 7f). In contrast, ATG5 deficiency obliterated the LC3 puncta induced by both Torin 1 and cGAMP.

**cGAMP activates ARF GTPases to regulate STING trafficking and signaling**

After budding from the ER, COP-II vesicles fuse with the cis-Golgi, and the cargo proteins further traffic to the trans-Golgi or form COP-I vesicles in a process known as retrograde transport, both of which require the ARF family of GTPases^12,13^. BFA and GCA inhibit ARF GTPases and blocked phosphorylation of TBK1 and IRF3 induced by DNA but not poly(I:C) (Extended Data Fig. 8e), suggesting that the ARF family is specifically involved in the DNA signaling pathway. To test if cGAMP stimulation activates ARF1, we treated BJ and HEK293T-STING cells with cGAMP and then immunoprecipitated GGA3, an effector protein known to bind specifically to GTP-bound ARF1. GGA3 associated with ARF1 after cGAMP stimulation, indicating that ARF1 was activated by cGAMP stimulation (Fig. 3e). siRNAs against ARF1 strongly inhibited the induction of IFNβ and CXCL10 by HT-DNA but not poly (I:C) in BJ cells (Extended Data Fig. 8f, g). Knockdown of ARF1 but not ARF4 also blocked STING translocation to the Golgi in Hela cells, whereas knockdown of ARF3, ARF5, or ARF6 partially inhibited STING trafficking (Extended Data Fig. 8h, i). These results suggest that the ARF family of GTPases, especially ARF1, is important for STING trafficking and signaling.

**Supplementary Tables**

**Table S1: Primers for qPCR.**

| **Genes** | **Forward Primers** | **Reverse Primers** |
| --- | --- | --- |
| IFN-β | CATTACCTGAAGGCCAAGGA | CAATTGTCCAGTCCCAGAGG |
| CXCL10 | GTGGCATTCAAGGAGTACCTC | TGATGGCCTTCGATTCTGGATT |
| TNFα | TGCTTGTTCCTCAGCCTCTT | GGTTTGCTACAACATGGGCT |
| SAR1A | TTGATCTTGGTGGGCACGAG | GGATTCCACGAGGCGAGAAT |
| ARF1 | CGTGGAAACCGTGGAGTACA | CGCTCTCTGTCATTGCTGTC |
| SEC24C | TGATGGTTGTGTCTGATGTGG | TGTCTCTGTTTCCCTTGTGTC |
| GAPDH | ACAGTCAGCCGCATCTTCTT | ACGACCAAATCCGTTGACTC |
| HSV-1(1) | CATCACCGACCCGGAGAGGGAC | GGGCCAGGCGCTTGTTGGTGTA |
| HSV-1(2) | TACAACCTGACCATCGCTTG | GCCCCCAGAGACTTGTTGTA |
| HSV-1(3) | CATCACCGACCCGGAGAGGGAC | GGGCCAGGCGCTTGTTGGTGTA |
| GAPDH | ATGACATCAAGAAGGTGGTG | CATACCAGGAAATGAGCTTG |

**Table S2: sgRNA sequences for CRISPR knock out.**

| **Genes** | **sgRNA sequence 1** | **sgRNA sequence 2** |
| --- | --- | --- |
| STING | GGATGTTCAGTGCCTGCGAG | AATATGACCATGCCAGCCCA |
| cGAS | CGATGGATCCCACCGAGTCT | AGGCTTCCGCACGGAATGCC |
| TBK1 | CATAAGCTTCCTTCGTCCAG | GAAGAACCTTCTAATGCCTA |
| ATG5 | TGATATAGCGTGAAACAAGT | TTCCATGAGTTTCCGATTGA |
| ATG9 | AGGATATTCGAGAGAAGAAG | AGGTTTTCAATATGGTGCCA |
| Beclin-1 | CCTGGACCGTGTCACCATCC | TCCTGGTTTCGCCTGGGCTG |
| ULK1 | GCACTCACCGTGCAGGTAGT | GACCTGGCCGACTACCTGCA |
| SEC24C | GTGTCACGAACAGCCTTCAC | GTGCCCGTAAGCTATCAATG |
| VPS34 | GAAACCGTTGTTCCTCCTAC | GGAACAACGGTTTCGCTCTT |
| WIPI2 | TTTTGCAGGTCCCTAGCTGT | TCGTCAGCCTTAAAGCACCA |
| GBF1 | CCATCGGGCATTTCGTTTGA | CCATCAAACGAAATGCCCGA |

**Table S3: siRNA sequences for knock-down experiments.**

| **Genes** | **siRNA sequence 1** | **siRNA sequence 2** |
| --- | --- | --- |
| Control | AAUUCUCCGAACGUGUCACGU |  |
| STING | GCCCGGAUUCGAACUUACAAU | GUCCAGGACUUGACAUCUUAA |
| SAR1A | CGUGAGAUAUUUGGGCUUUAU | GAAUCCAAAGUUGAGCUUAAU |
| SEC24C | ACUUAUGUUAUCGAGUCAAUG | UUGAUGUAAAGCGACUAAUAU |
| ARF1 | CCAUUCCCACCAUAGGCUU | CACCAUAGGCUUCAACGUGGA |
| ARF3 | CAAGAGCCUGAUUGGGAAGAA |  |
| ARF4 | CCAUCAGUGAAAUGACAGAUA |  |
| ARF5 | UGCAUGUUCUCUCUGUUGUUG |  |
| ARF6 | AGCUGCACCGCAUUAUCAAUG |  |

**References**

1 Liu, S. *et al.* Phosphorylation of innate immune adaptor proteins MAVS, STING, and TRIF induces IRF3 activation. *Science* **347**, aaa2630, doi:10.1126/science.aaa2630 (2015).

2 Liu, Y. *et al.* Activated STING in a vascular and pulmonary syndrome. *N Engl J Med* **371**, 507-518, doi:10.1056/NEJMoa1312625 (2014).

3 Davies, B. W., Bogard, R. W., Young, T. S. & Mekalanos, J. J. Coordinated regulation of accessory genetic elements produces cyclic di-nucleotides for V. cholerae virulence. *Cell* **149**, 358-370, doi:10.1016/j.cell.2012.01.053 (2012).

4 Gutierrez, M. G., Munafo, D. B., Beron, W. & Colombo, M. I. Rab7 is required for the normal progression of the autophagic pathway in mammalian cells. *J Cell Sci* **117**, 2687-2697, doi:10.1242/jcs.01114 (2004).

5 Jager, S. *et al.* Role for Rab7 in maturation of late autophagic vacuoles. *J Cell Sci* **117**, 4837-4848, doi:10.1242/jcs.01370 (2004).

6 Kim, J. *et al.* Differential regulation of distinct Vps34 complexes by AMPK in nutrient stress and autophagy. *Cell* **152**, 290-303, doi:10.1016/j.cell.2012.12.016 (2013).

7 Russell, R. C. *et al.* ULK1 induces autophagy by phosphorylating Beclin-1 and activating VPS34 lipid kinase. *Nat Cell Biol* **15**, 741-750, doi:10.1038/ncb2757 (2013).

8 Dooley, H. C. *et al.* WIPI2 links LC3 conjugation with PI3P, autophagosome formation, and pathogen clearance by recruiting Atg12-5-16L1. *Mol Cell* **55**, 238-252, doi:10.1016/j.molcel.2014.05.021 (2014).

9 Jaber, N. *et al.* Class III PI3K Vps34 plays an essential role in autophagy and in heart and liver function. *Proc Natl Acad Sci U S A* **109**, 2003-2008, doi:10.1073/pnas.1112848109 (2012).

10 He, R., Peng, J., Yuan, P., Xu, F. & Wei, W. Divergent roles of BECN1 in LC3 lipidation and autophagosomal function. *Autophagy* **11**, 740-747, doi:10.1080/15548627.2015.1034404 (2015).

11 Devereaux, K. *et al.* Regulation of mammalian autophagy by class II and III PI 3-kinases through PI3P synthesis. *PLoS One* **8**, e76405, doi:10.1371/journal.pone.0076405 (2013).

12 D'Souza-Schorey, C. & Chavrier, P. ARF proteins: roles in membrane traffic and beyond. *Nat Rev Mol Cell Biol* **7**, 347-358, doi:10.1038/nrm1910 (2006).

13 Brandizzi, F. & Barlowe, C. Organization of the ER-Golgi interface for membrane traffic control. *Nat Rev Mol Cell Biol* **14**, 382-392, doi:10.1038/nrm3588 (2013).
